# *BigSeqKit*: a parallel Big Data toolkit to process FASTA and FASTQ files at scale

**DOI:** 10.1093/gigascience/giad062

**Published:** 2023-07-31

**Authors:** César Piñeiro, Juan C Pichel

**Affiliations:** CiTIUS, Universidade de Santiago de Compostela, Santiago de Compostela 15782, Spain; CiTIUS, Universidade de Santiago de Compostela, Santiago de Compostela 15782, Spain

**Keywords:** FASTA/FASTQ files, Performance, Parallelism, Big Data

## Abstract

**Background:**

High-throughput sequencing technologies have led to an unprecedented explosion in the amounts of sequencing data available, which are typically stored using FASTA and FASTQ files. We can find in the literature several tools to process and manipulate those type of files with the aim of transforming sequence data into biological knowledge. However, none of them are well fitted for processing efficiently very large files, likely in the order of terabytes in the following years, since they are based on sequential processing. Only some routines of the well-known *seqkit* tool are partly parallelized. In any case, its scalability is limited to use few threads on a single computing node.

**Results:**

Our approach, *BigSeqKit*, takes advantage of a high-performance computing–Big Data framework to parallelize and optimize the commands included in *seqkit* with the aim of speeding up the manipulation of FASTA/FASTQ files. In this way, in most cases, it is from tens to hundreds of times faster than several state-of-the-art tools. At the same time, our toolkit is easy to use and install on any kind of hardware platform (local server or cluster), and its routines can be used as a bioinformatics library or from the command line.

**Conclusions:**

*BigSeqKit* is a very complete and ultra-fast toolkit to process and manipulate large FASTA and FASTQ files. It is publicly available at https://github.com/citiususc/BigSeqKit.

## Introduction

The history of modern DNA sequencing started several decades ago and, since then, has seen astounding growth in sequencing capacity and speed. From the first genomes with a few thousand bases, DNA sequencing has advanced to sequence the human genome of 3 billion bases. In recent years, next-generation sequencing (NGS) technology, also known as massive parallel sequencing (MPS), has made it possible to expand the amount of sequencing data available. For example, the Illumina NovaSeq 6000 [1] platform can generate a maximum output of 6 Tb of data and read about 20 billion sequences per run. Note that sequences, commonly named *reads*, are composed of ASCII characters representing a nucleotide (base) from the sequence. In the DNA case, we can only find 4 possible bases (A—adenine, C—cytosine, G—guanine, and T—thymine).

**Figure 1:**
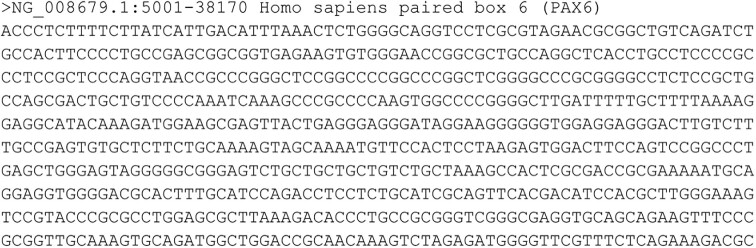
Example of FASTA file showing the first part of the PAX6 gene (obtained from [4]).

**Figure 2:**
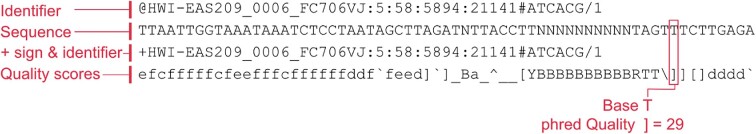
Example of FASTQ file format (obtained from [4]).

The NGS raw data are mainly stored in FASTA [2] and FASTQ [3] text-based file formats. In particular, nucleotide and protein sequences are typically stored in the FASTA file format, whereas FASTQ is the most widely used format for sequencing read data. An example of FASTA file is shown in Fig. 1. A sequence in FASTA format begins with a single-line description about the sequence in the subsequent lines. The description line is distinguished from the sequence data by a greater-than (>) symbol at the beginning. On the other hand, the FASTQ format was designed to handle the quality metrics of the sequences obtained from the sequencers. In FASTQ, every 4 lines describe a sequence or read. An example is displayed in Fig. 2. The information provided per read is as follows: identifier and an optional description (first line), sequence (second line), and the quality score of the read (fourth line). An extra field, represented by symbol “+,” is used as separator between the data and the quality information (third line).

Manipulating these files efficiently is essential to analyze and interpret data in any genomics pipeline. Common operations on FASTA and FASTQ files include searching, filtering, sampling, deduplication, and sorting, among others. We can find several tools in the literature for FASTA/Q file manipulation such as *HTSeq* [5], *FASTX* [6], *fqtools* [7], *seqtk* [8], Biopython [9], samtools [10], *pyfadix* [11], *pyfastx* [12], and *seqkit* [13]. These tools can be classified according to how the sequences are parsed [12]. In the first category, sequences are processed in order, which causes important overheads when extracting and randomly sampling sequences. That is the case of *HTSeq, FASTX, fqtools*, and *seqtk*. In the second category, we find tools that support random access to sequences by establishing an index file. Tools belonging to this category are more efficient in terms of performance and memory consumption. However, none of them are well fitted for processing very large files of hundreds of GB (likely TBs in the near future) since they are based on sequential processing. The exception is *seqkit* that allows some routines to use a few threads, but in any case, its scalability is very limited.

To deal with this issue, in this article, we introduce *BigSeqKit*, a parallel toolkit to manipulate FASTA and FASTQ files at scale with speed and scalability at its core. *BigSeqKit* takes advantage of IgnisHPC [14, 15], a computing engine that unifies the development, combination, and execution of high-performance computing (HPC) and Big Data parallel tasks using different languages and programming models. As it was demonstrated, IgnisHPC outperforms the state-of-the-art framework Spark [16] in terms of performance and scalability running applications that represent the most typical algorithmic patterns in Big Data and scientific computing.

*BigSeqKit* uses the *seqkit* routines as basis since that toolkit covers a wide range of utilities and is one of the most used by the bioinformatics research community. As a consequence, *BigSeqKit* will offer the same functionalities and command interface [17]. *BigSeqKit* can be used from the command line, but it is at the same time a library, so its routines can also be called from a C/C++, Python, Go, or Java application.

Another important characteristic of *BigSeqKit* is that it is fully containerized, which isolates the execution environment from the physical system and avoids dependency problems. As a consequence, *BigSeqKit* is very easy to install and can run on a local server or on any type of cluster since it supports some of the most important resource and scheduler managers (e.g., Mesos [18], Nomad [19], and Slurm [20]).

## Background

IgnisHPC [14, 15] unifies the execution of Big Data and HPC workloads in the same computing engine. Unlike other frameworks such as Hadoop [21] and Spark [16], IgnisHPC has native support for multilanguage applications using both JVM (Java Virtual Machine) and non-JVM-based languages. In this way, applications can be implemented using 1 or several programming languages following an API inspired by Spark’s one.

The previous version of IgnisHPC supported natively C, C++, Java, and Python. However, *seqkit* was implemented using the Go programming language. Since *BigSeqKit* parallelizes and optimizes the *seqkit* routines using IgnisHPC, it was necessary to add support for this language in the framework. Another solution would require to port the complete toolkit to a different language, which is a difficult task prone to errors. It is worth noting that, to the best of our knowledge, nowadays IgnisHPC is the first parallel computing framework to include native support for this language. Considering Spark instead of IgnisHPC is not an option because, as it was demonstrated in [14], when using a nonnative language code, data transfers between the JVM and external processes degrade noticeably Spark’s overall performance.

Go is a programming language with a simple syntax that was designed to be easy to learn and use. With the release of Go v1.18, the language included support for Generics, which allows the creation of functions, types, and methods that can work with any data type. This makes Go an effective and user-friendly way to implement Big Data interfaces. The implementation of Go in IgnisHPC is similar to that of C++, as both are compiled and statically typed languages. However, Go replaces the concept of inheritance with composition, which does not change the philosophy of use in IgnisHPC. Big Data functions are still accessible through the IgnisHPC API, and users can create their own code by implementing the same interfaces.

One of the key features of IgnisHPC is its use of containers to isolate and execute code. Containers are lightweight and portable, making it easy to run IgnisHPC on a variety of different clusters, including both HPC and Big Data. IgnisHPC is also tolerant to failures, as the containers or processes can be easily restarted if there are issues. In particular, if some data are lost, IgnisHPC has enough information about how it was derived. In this way, only those operations needed to recompute the corresponding portion of data are performed.

We must highlight that although the IgnisHPC API [22] uses a sequential notation, operations on data are performed in parallel. As we pointed out, the IgnisHPC API was inspired by the Spark API in such a way that IgnisHPC codes are easily understandable by users who are familiar with Spark. Table 1 shows a list of some of the most important functions supported by IgnisHPC. In particular:

- *Map functions:* The common characteristic to routines belonging to this type is that they apply the same function to each element in the data. As a result of the transformation, the output could be of different size with respect to the input.
- *Reduce functions:*  reduce and treeReduce methods aggregate all the elements in the input data using a function. aggregate and treeAggregate are a sort of reduction where the type of the input and output data is different. In this case, 2 functions are necessary; the first one is applied to each element in a data partition, and the second one combines the partial results obtained for each partition. reduceByKey and aggregateByKey are variations where the operation is performed only among elements with the same key in such a way that the final result is a set of unique pairs with values calculated using reduce or aggregate operations, respectively.
- *Group functions:* These methods group elements in a data frame according to their key value (groupByKey) or a user-defined function (groupBy).
- *Sort functions:* In order to sort elements, IgnisHPC provides 3 functions: sort, sortByKey, and sortBy. The first method uses the natural order and does not need any additional function. sortByKey sorts the keys using their natural order. sortBy allows to use a user-defined function to specify the order of the elements. If the result of applying that function to 2 elements is *true*, then the first element should precede the second one. All methods support ascending and descending order.
- *SQL functions:* These functions operate on data frames. union concatenates 2 data frames, join merges elements of 2 data frames whose keys match, and distinct returns a new data frame after removing the duplicate records. These methods are necessary, for example, in many graph processing problems.
- *Other functions:* IgnisHPC implements several operations that return a value to the driver code, but they do not modify or generate new stored data. Spark refers to this type of operations as *actions*. For instance, IgnisHPC supports methods such as count, take, takeSample, and collect. The most basic operation is count that returns the number of elements of a stored data collection. collect returns a collection with all the elements stored in the executors of a task. take applies a collect operation but obtains only the first *n* elements, where *n* is chosen by the user. takeSample returns a random sample of *n* elements from the distributed data, with or without replacement. Finally, another interesting routine is parallelize, which distributes the elements of a collection among the executors to form a distributed dataset. In this case, new stored data are created.

**Table 1:** Some of the most important IgnisHPC API functions

Type	Functions
Map	map, flatmap, mapWithIndex, filter, keyBy, keys, values, mapPartitions, mapValues, etc.
Reduce	reduce, treeReduce, aggregate, treeAggregate, reduceByKey, aggregateByKey, etc.
Group	groupBy, groupByKey
Sort	sort, sortBy, sortByKey
I/O	parallelize,collect, top, take, saveAsObjectFile, saveAsTextFile, saveAsJsonFile, etc
SQL	union, join, distinct
Math	sample, sampleByKey, take, takeSample, count, countByKey, countByValue, max, min, etc.
Balancing	repartition, partitionByHash, partitionByRandom, partitionBy
Persistence	persist, cache, unpersist, uncache

It is worth noting that the IgnisHPC API functions allow users to parallelize a code with a high level of abstraction. In this way, it is only necessary to focus on data dependencies.

## Methods

As we commented previously, *BigSeqKit* (RRID:SCR_023592) speeds up the *seqkit* routines through parallelization and optimization techniques. Table 2 shows the routines supported by the current version of *BigSeqKit*. Despite most of the commands in *seqkit* are sequential, we can classify each command implementation into 3 categories according to its inherent parallelism:

- Independent: it is a embarrassingly parallel workload. As a consequence, the computation could be applied to all sequences in parallel. An example is seq, a function that transforms sequences. In this case, the transformation only affects each sequence individually.
- Partially dependent: computations could be done in parallel, but the method requires some type of consensus to obtain the result. For instance, stats should merge the partial results computed for each sequence to calculate some statistics of the considered FASTA/Q file.
- Dependent: dependencies between sequences prevent the method from being executed in parallel. As a consequence, *BigSeqKit* requires a complete new algorithm to perform the same command in parallel. rmdup is a good example because with the aim of removing duplicated sequences, it is necessary to read all of them before generating a result.

**Table 2:** List of commands included in both *BigSeqKit* and *seqkit*. Those commands with an asterisk support new functionalities not included in *seqkit*

**Basic commands**	
seq	Transform sequences (extract ID, filter by length, remove gaps, reverse complement, etc.)
subseq	Get subsequences by region/gtf/bed, including flanking sequences
stats	Simple statistics of FASTA/Q files: #seqs, min/max length, N50, Q20%, Q30%, etc.
faidx*	Create FASTA or FASTQ index file and extract subsequences
**Format conversion**	
fa2fq	Retrieve corresponding FASTQ records by a FASTA file
fq2fa	Convert FASTQ file to FASTA format
translate	Translate DNA/RNA to protein sequence
**Searching**	
grep	Search sequences by ID/name/sequence/sequence motifs
locate	Locate subsequences/motifs
**Set operations**	
sample	Sample sequences by number or proportion
rmdup	Remove duplicated sequences by ID/name/sequence
common	Find common sequences of multiple files by ID/name/sequence
duplicate	Duplicate sequences *N* times
head	Print first *N* FASTA/Q records
head-genome	Print sequences of the first genome with common prefixes in name
pair	Match up paired-end reads from 2 FASTQ files
range	Print FASTA/Q records in a range (start:end)
**Edit**	
concat	Concatenate sequences with the same ID from multiple files
replace	Replace name/sequence using a regular expression
rename	Rename duplicated IDs
**Ordering**	
sort	Sort sequences by ID/name/sequence/length
shuffle	Shuffle sequences

The integration, parallelization, and optimization of each *seqkit* command in IgnisHPC will be different depending on its category. More details are provided below.

### Independent routines

For these commands, the computation can be applied to all sequences in parallel because there are no dependencies (communication) among them. In other words, routines belonging to this category can be processed using an embarrassingly parallel approach. Considering the IgnisHPC (and Spark) API, it is only necessary to use map functions to parallelize the computations. As we pointed out, the common characteristic to these API functions is that they apply the same operation to each element in the data.

The following *BigSeqKit* commands belong to this category: seq, subseq, stats, fq2fa, fa2fq, translate, grep, locate, duplicate, and replace (see Table 2 for details).

### Partially dependent routines

As we mentioned, this category includes commands in which computations can be done in parallel using map functions, but the methods require some type of consensus to get the desired outcome. This consensus can be easily implemented using the IgnisHPC API. The following *BigSeqKit* commands belong to this category:

- stats: statistics can be generated in parallel but the final result must be unique, so all partial results must be merged using a reduction (reduce operation in the IgnisHPC API).
- head: sequences should know their position inside the file to check if they are inside the head window. To do that, it is necessary to use mapWithIndex, a special map operation included in the IgnisHPC API that allows each element to know its global index within a data structure.
- head-genome: similar to head, but not all sequences are valid. In order to determine the window, invalid sequences must be removed first.
- range: also similar to head. Sequences should know their position inside the file to check if they are within the range window.
- grep: although this command was included in the previous category, a command option (–delete-matched) limits the number of results to just 1 per search pattern. In such cases, it is necessary to remove the extra results.
- faidx: also similar to head, sequences compute their offsets inside the input file using mapPartitionWithIndex and exchange the information between executors to perform a parallel indexing operation with a simple map.

### Dependent routines

Commands belonging to this category have an implementation in *seqkit* that by its nature cannot be parallelized. However, IgnisHPC allows us to define the implementation at a high level, which increases noticeably the productivity. Behaviors and functionalities will be preserved in *BigSeqKit* but through a complete new parallel implementation. In particular:

- sample: a sequential sampling can be performed in parallel if we split the sequences and run a sample for each partition. It was mathematically proven that sampling without replacement follows a hypergeometric function [23]. In this way, we can calculate the proportion of the sample that corresponds to each partition.
- rmdup: sequences are grouped (groupBy API function) using a hash with the ID, name, or sequence. In those groups containing more than 1 element, a search for duplicates is carried out to remove them.
- pair and concat: sequences of the input files generate key–value pairs where the key is the ID and the value is the sequence with its index file (map). Pairs are unified by means of union and grouped using groupByKey. Afterward, sequences in the same group are paired or concatenated if they belong to different files.
- common: the first stage of the command is the same one explained above for pair and concat. Then if a sequence can be found in all files, we check its index file, to avoid its deletion.
- rename: sequences are grouped (groupBy) using their ID, and then IDs in the same group are renamed.
- sort: the sequential sort algorithm implemented in *seqkit* is replaced by a sample MergeSort [24] algorithm that can be efficiently executed in parallel in a distributed environment.
- shuffle: sequences shuffling can be implemented using the IgnisHPC API function partitionByRandom.

### Another implementation details

In order to parallelize and integrate the *seqkit* routines into IgnisHPC, it was necessary to start considering the sequence parser. It takes a stream of characters in FASTA and FASTQ format and generates a data structure with the sequence representation. In *seqkit*, this stream can be represented by a file or the standard input. In *BigSeqKit*, this stream is implemented using the IgnisHPC iterators, which grant the users access to the data partitions. In this way, *BigSeqKit* will read the data from a file and split it in multiple partitions, which facilitates their parallel processing. In particular, each worker reads a portion of the input file, so the input/output (I/O) operation is performed in parallel. There is 1 worker per computing node. Within each worker, its portion of the file is further divided among the available threads, improving the overall I/O performance. As a result, the *seqkit* command arguments that affect file processing will have no effect in *BigSeqKit*. For example, the –two-pass option, which reads a file multiple times instead of storing all the sequences in memory, does not make sense in *BigSeqKit*. We must highlight that the fact of splitting the input files between several computing nodes in *BigSeqKit* means that the memory consumed by node is also split, which allows our tool to work with larger datasets. In addition, *BigSeqKit* also reduces the memory footprint by only storing the IDs and indices of each sequence.

Another important advantage of using IgnisHPC is how memory is handled. Users can choose a type of storage according to their particular case. For instance, if an input file is too large to be kept completely in the server memory, it could be stored compressed in memory or in disk. Performance would be lower, but it could be successfully processed. That scenario is not considered by *seqkit* that simply would raise an “out of memory” error. In particular, *BigSeqKit* supports the following storage options:

- *In-Memory:* it is the best performer since all data are stored in memory. It is the default option.
- *Raw memory:* data are stored in a memory buffer using a serialized binary format. Extra memory consumption is minimal and the buffer is compressed by Zlib.
- *Disk:* similar to raw memory but the buffer is stored as a POSIX file. Although the performance is significantly worse, it enables working with vast amounts of data that cannot be entirely kept in memory.

On the other hand, rmdup, common, and pair commands in *seqkit* use hash functions to check duplicates. It is well known that hash functions can produce the same result for different values. This event is commonly known as a hash collision. However, *seqkit* does not check for collisions, so it is possible to generate incorrect results. *BigSeqKit* uses hashes to group sequences but then checks for collisions by comparing the real values.

Finally, *seqkit* and other state-of-the-art tools build index files (faidx routine) to speed up some other tasks (e.g., searches). Although *BigSeqKit* is also capable of creating those index files, it does not require them to improve its performance since data within IgnisHPC are already indexed. In other words, the index is created while reading the input file.

### New functionalities

*BigSeqKit* not only enables the parallelization of *seqkit* functions but also improves its algorithms to provide benefits even for sequential executions and includes additional functionalities. In particular, the faidx command in *seqkit* implements indexing of FASTA files using the *samtools* format, but FASTQ files are not supported. *BigSeqKit* adds support for this type of files and generates an index file using the *samtools* format as well. Note that this is the most widespread format and is also supported by other state-of-the-art tools. Therefore, *BigSeqKit* allows indexing of both FASTA and FASTQ files using the same syntax than *seqkit*.

## How to Use *BigSeqKit*

*BigSeqKit* can be used in 2 different ways. The first one is by means of a command-line interface (CLI). This approach is similar to the “command subcommand” structure adopted by *seqkit* [13]. In this way, it is only necessary to select a subcommand or routine (see a complete list in Table 2) and pass its arguments through command line. As we mentioned previously, to improve the usability and facilitate the adoption of *BigSeqKit*, it implements the same command interface as *seqkit*.

Since *BigSeqKit* runs within the IgnisHPC framework, it is necessary to send the *BigSeqKit* routine through the IgnisHPC submitter. For instance, if we are running *BigSeqKit* on a local server, the following expression uses the routine *seq* to print the name of the sequences included in a FASTA file to an output file:


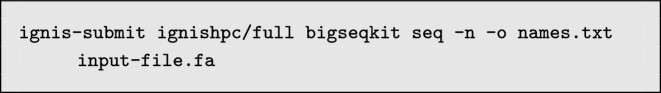


Therefore, the syntax should be: ignis-submit ignishpc/full bigseqkit <cmd> <arguments>.

In addition, users can also specify through arguments the number of instances, cores, and memory (in GB) to be used in the execution. By default, those values are set to 1. For example, we can execute the previous command using 2 cores:


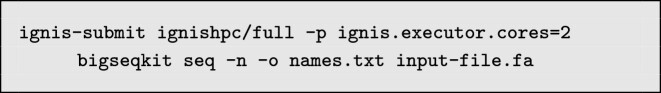


Unlike the other state-of-the-art tools, *BigSeqKit* can also be executed on a parallel cluster. Typical HPC clusters have Slurm [20] as the preferred resource manager and Singularity [25] as a container-based technology. In this case, users will send the job using the ignis-slurm submitter instead of ignis-submit.

**Figure 3:**
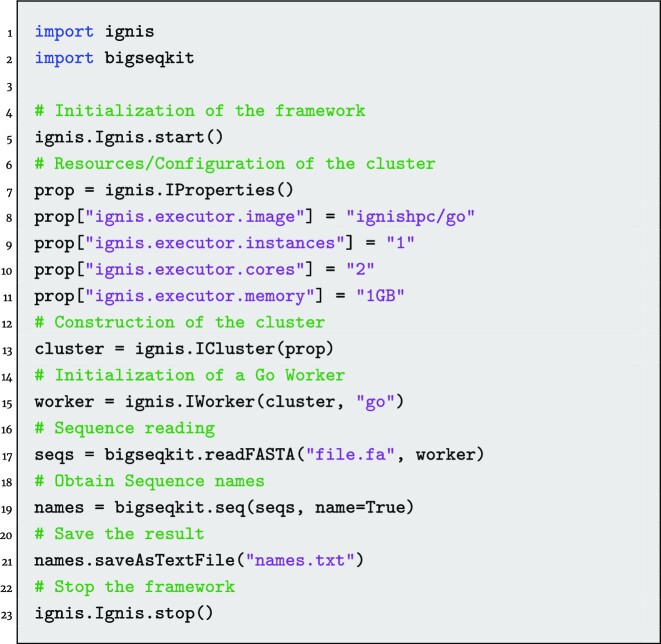
Example of Python code using the *BigSeqKit* routines.

On the other hand, *BigSeqKit* can also be used as a bioinformatics library. It is worth noting that *BigSeqKit* was implemented in Go language. However, thanks to the multilanguage support provided by IgnisHPC, it is possible to call *BigSeqKit* routines from C/C++, Python, Java, and Go applications without additional overhead. An example of Python code is shown in Fig. 3. This example is equivalent to the previous one used in the explanation of the CLI. Since *BigSeqKit* has been created as a library, it only needs to be imported to be used. Functions in *BigSeqKit* do not use files as input; they use DataFrames instead, an abstract representation of parallel data used by IgnisHPC (similar to RDDs in Spark). Parameters are grouped in a data structure where each field represents the long names of a parameter. We must highlight that *BigSeqKit* functions can be linked (like system pipes using “|”), so the DataFrame generated by one can be used as input to another. In this way, integrating *BigSeqKit* routines in a more complex code is really easy.

The code starts initializing the IgnisHPC framework (line 5 in Fig. 3). Next, a cluster of containers is configured and built (lines from 7 to 15). Multiple parameters can be used to configure the environment such as image, number of containers, number of cores, and memory per container. In this example, we will use 1 node (instances) and 2 cores by node. After configuring the IgnisHPC execution environment, the *BigSeqKit* code actually starts. First, we read the input file (line 17). There is a different function for reading FASTA and FASTQ files. All the input sequences are stored as a single data structure. The next stage consists of printing the name of the sequences included in the FASTA file (line 19). The function takes as parameters the sequences and the options that specify its behavior. Finally, the names of the sequences are written to disk. It is important to highlight that lazy evaluation is performed, so functions are only executed when the result is required to be saved on disk.

## Experimental Results

In this section, we analyze the performance results obtained by *BigSeqKit* with respect to other state-of-the-art tools. In particular, we have considered *samtools, pyfastx*, and *seqkit* for their performance and number of commands supported. Experiments were conducted using up to 8 computing nodes of the FinisTerrae III [26] supercomputer installed at CESGA (Spain). Each node contains a 32-core Intel Xeon Ice Lake 8352Y @2.2 GHz processor and 256 GB of memory interconnected with Infiniband HDR 100. It is a Linux cluster running Rocky Linux v8.4 (kernel v4.18.0). We have used SingularityCE v3.9.7 (containers), IgnisHPC v2.2, *pyfastx* v0.8.4, *samtools* v1.16.1, and *seqkit* v2.3.1 (with Slurm as cluster manager and Lustre as distributed file system).

The performance evaluation was carried out using as input 6 different FASTA/FASTQ files that cover a wide variety of characteristics and sizes. The main features of these files are the following:

- *D*_1_ (m64013e_210227_222017.hifi_reads—FASTA—24 GB):
  Number of sequences: 1.2M, Minimum length: 85, Average length: 19.7K, Maximum length: 48.5K.
- *D*_2_ (SRR642648_1.filt—FASTQ—24.1 GB):
  Number of sequences: 98.7M, Minimum length: 100, Average length: 100, Maximum length: 100.
- *D*_3_ (Homo_sapiens.GRCh38.dna_sm.toplevel—FASTA—59.7 GB):
  Number of sequences: 639, Minimum length: 970, Average length: 98.8M, Maximum length: 248.9M.
- *D*_4_ (ERR4667750—FASTQ—79.1 GB):
  Number of sequences: 318.1M, Minimum length: 101, Average length: 101, Maximum length: 101.
- *D*_5_ (uniprot_trembl—FASTA—104 GB):
  Number of sequences: 229.9M, Minimum length: 7, Average length: 351.6, Maximum length: 45.3K.
- *D*_6_ (DRR002180_2—FASTQ—395 GB):
  Number of sequences: 1.625B, Minimum length: 101, Average length: 101, Maximum length: 101.

As example to illustrate the benefits of our tool, we will evaluate the following utilities (see Table 2 for a complete list of commands): faidx builds an index for FASTA/FASTQ files, locate locates sequences following some search pattern, replace replaces a name/sequence using a regular expression, rmdup removes duplicated sequences, sample selects sequences by number or proportion, seq transforms sequences (extract ID, filter by length, etc.) and removes gaps, and sort sorts sequences by ID/name/sequence/length. We will also include the performance results of the corresponding utilities, if they exist, for *samtools, pyfastx*, and *seqkit*. Execution times for all the tools considered include the overhead of loading sequences into memory and the subsequent writing of results to disk. Note that the “2-pass” argument of *seqkit* was not used in the experiments. Each result was computed as the median of 5 experiments. For the sake of reproducibility, all the codes and scripts used for performing the benchmarks are freely available at the *BigSeqKit* repository.

**Figure 4:**
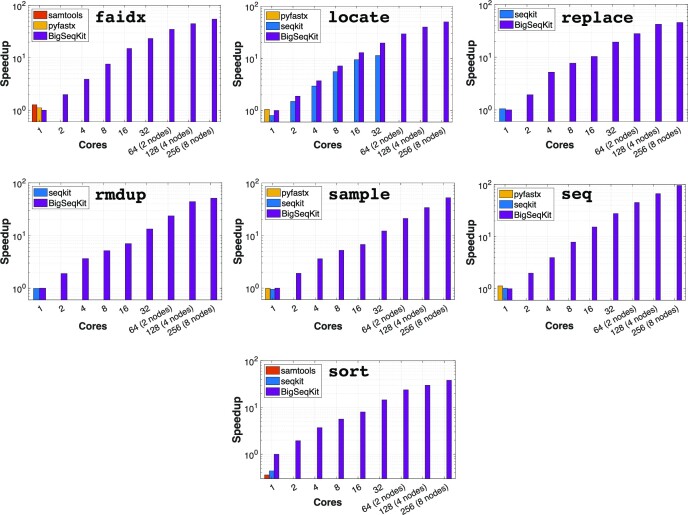
Speedups (in log scale) obtained by *BigSeqKit* and other state-of-the-art tools with respect to the *BigSeqKit* sequential time when executing different commands using D_4_ as input. Note that locate was parallelized in *seqkit*.

First, in order to provide an overall idea about the scalability and performance of *BigSeqKit* with respect to the other state-of-the-art tools, we will show the speedups obtained for the D_4_ dataset using different number of cores. The behavior is very similar when considering the other datasets. Results in log scale are displayed in Fig. 4. Speedups were calculated using as reference the sequential execution (1 core) of the corresponding *BigSeqKit* command. According to the results, several conclusions can be made. It can be observed that the scalability of *BigSeqKit* is quite good, reaching speedups up to 27.7× and 95.7× (seq command) using 1 server (32 cores) and 8 computing nodes (256 cores), respectively. Note that speedups of some routines are not higher when using 256 cores due to a small fraction of the code that should be executed sequentially (Amdahl’s law).

While *samtools* and *pyfastx* routines are always processed sequentially, *seqkit* uses a multithreaded approach to (partly) parallelize some commands. However, its scalability is limited to use a few threads on a single server (computing node). This is the case of locate. Its best speedup only reaches 11.3× (32 cores) while this value increases until 19.6× with *BigSeqKit*. If 8 nodes are used, *BigSeqKit* is 49.9× faster than the sequential execution.

For all the commands studied, *BigSeqKit* clearly outperforms *samtools, pyfastx*, and *seqkit*. There are only a few cases using 1 core where the speedups of these tools are slightly greater than 1 (e.g., executing the faidx routine with *samtools* and *pyfastx*). However, other commands such as sort and sample are processed faster with *BigSeqKit* even using 1 core.

Tables from 3 to 9 display, for all the datasets, the execution times of *BigSeqKit* and the other state-of-the-art tools when running faidx, locate, replace, rmdup, sample, seq, and sort utilities, respectively. Speedups with respect to the sequential execution of the corresponding *BigSeqKit* command are shown between brackets. Highlighted is the fastest time overall and the corresponding speedup. Note that *BigSeqKit* stores compressed in memory the largest dataset D_6_ when using 1 computing node since it exceeds the memory capacity of an individual server (see the *Raw memory* storage option in the Background section).

For all the experiments conducted, *BigSeqKit* is always the fastest tool both considering a single server (1 node) or several computing nodes. In any case, let’s take a look in detail of the behavior for each command:

- faidx (Table 3): *BigSeqKit* speedups range from 5.4× to 27.4× considering a single server (32 cores) and from 7.2× to 144× with 8 nodes. It means, for example, building the index file for our largest dataset D_6_ (395 GB) in just 5.8 minutes (single server), while *samtools* and *pyfastx* require about 2.1 hours. This time decreases to 1 minute when *BigSeqKit* uses 8 nodes. As mentioned previously, the faidx routine in *seqkit* does not support FASTQ files (D_2_, D_4_, and D_6_).
- locate (Table 4): the searching routines, grep and locate, are very expensive in terms of computations. Note that considering sequential processing, locate takes more than 3 hours to process our smallest dataset D_1_ independently of the tool considered. This time increases to more than 3 days of computation for D_6_. *seqkit* has a multithread version of locate, which obtains speedups from 10.5× to 18.8×. These speedups are always lower than the ones obtained by *BigSeqKit* on a single server. It is important to highlight that *seqkit* raises an out-of-memory error when processing D_6_ with 1, 2, and 4 cores. On the other hand, when using 8 nodes, *BigSeqKit* achieves noticeable speedups up to 104.1×. In this way, it is able to reduce the time necessary to execute the locate command with our largest dataset D_6_ from 3 days to only 0.8 hours.
- replace (Table 5): this routine (or an equivalent) is not supported by *samtools* and *pyfastx*. In this case, *BigSeqKit* is from tens to hundreds of times faster than *seqkit*, reaching speedups up to 159.8×.
- rmdup (Table 6): this routine is also not supported by *samtools* and *pyfastx*. In this case, *BigSeqKit* is tens of times faster than *seqkit*, achieving a maximum speedup of 74.7× when removing the duplicated sequences in D_5_.
- sample (Table 7): operation not supported by *samtools BigSeqKit* is again faster than the other tools, increasing the speedups as the input data size grows. It can be observed that *BigSeqKit* is able to sample sequences in seconds. For instance, *pyfastx* and *seqkit* take about 3 hours to process D_6_, while *BigSeqKit* requires just 2 minutes.
- seq (Table 8): operation not supported by *samtools*. Performance results are similar to the sample ones in such a way that *BigSeqKit* filters sequences by ID in a few seconds, achieving a noticeable speedup of 169.7×. It should be noted that among the routines examined in this study, seq is the least computationally demanding.
- sort (Table 9): this routine was not included in *pyfastx*. In general, the performance of *samtools* and *seqkit* is poor. And, most importantly, both tools produce memory errors when processing the largest dataset D_6_, so it cannot be sorted. However, *BigSeqKit* sorts D_6_ 21.8× and 131.1× faster than the sequential version using a single server and 8 computing nodes, respectively. It means that the time decreases from 5 hours to barely 2 minutes.

**Table 3:** Execution times (seconds) using different number of cores: faidx command. Highlighted are fastest time and number of times faster than sequential *BigSeqKit*

	1	2	4	8	16	32	64 (2 nodes)	128 (4 nodes)	256 (8 nodes)
**D_1_**									
*samtools*	86.2 [1.03×]	–	–	–	–	–	–	–	–
*pyfastx*	109.2 [0.81×]	–	–	–	–	–	–	–	–
*seqkit*	75.4 [1.17×]	–	–	–	–	–	–	–	–
*BigSeqKit*	88.4	46.0	35.3	26.3	19.4	16.3 [5.4×]	13.6	**12.3 [7.2×]**	12.5
**D_2_**									
*samtools*	165.6 [1.06×]	–	–	–	–	–	–	–	–
*pyfastx*	177.9 [0.99×]	–	–	–	–	–	–	–	–
*BigSeqKit*	175.9	90.8	67.4	50.3	39.1	31.4 [5.6×]	23.4	19.1	**15.5 [11.3×]**
**D_3_**									
*samtools*	210.0 [0.77×]	–	–	–	–	–	–	–	–
*pyfastx*	131.2 [1.23×]	–	–	–	–	–	–	–	–
*seqkit*	131.8 [1.23×]	–	–	–	–	–	–	–	–
*BigSeqKit*	161.9	83.9	61.7	24.5	17.5	15.7 [10.3×]	13.6	**13.4 [12.1×]**	14.7
**D_4_**									
*samtools*	538.4 [1.27×]	–	–	–	–	–	–	–	–
*pyfastx*	615.5 [1.11×]	–	–	–	–	–	–	–	–
*BigSeqKit*	684.2	346.6	175.2	90.3	45.4	29.3 [23.3×]	19.6	15.3	**12.5 [54.7×]**
**D_5_**									
*samtools*	771.0 [1.08×]	–	–	–	–	–	–	–	–
*pyfastx*	634.3 [1.31×]	–	–	–	–	–	–	–	–
*seqkit*	1,096.2 [0.76×]	–	–	–	–	–	–	–	–
*BigSeqKit*	829.8	361.3	179.4	89.3	49.4	30.3 [27.4×]	23.6	19.3	**16.5 [50.3×]**
**D_6_**									
*samtools*	7,651.6 [1.14×]	–	–	–	–	–	–	–	–
*pyfastx*	7,712.5 [1.13×]	–	–	–	–	–	–	–	–
*BigSeqKit*	8,712.3	4,423.3	2,282.2	1,191.9	640.2	350.4 [24.9×]	129.5	85.3	**60.5 [144×]**

**Table 4:** Execution times (seconds) using different number of cores: locate command. Highlighted are fastest time and number of times faster than sequential *BigSeqKit*

	1	2	4	8	16	32	64 (2 nodes)	128 (4 nodes)	256 (8 nodes)
**D_1_**									
*pyfastx*	11,523.5 [1.0×]	–	–	–	–	–	–	–	–
*seqkit*	12,822.9	6,385.0	3,210.9	1,731.4	940.5	612.4 [18.8×]	–	–	–
*BigSeqKit*	11,486.2	6,286.1	3,180.0	1,637.3	850.9	470.6 [24.4×]	264.6	156.9	**110.3 [104.1×]**
**D_2_**									
*pyfastx*	8,841.2 [1.2×]	–	–	–	–	–	–	–	–
*seqkit*	12,319.8	6,909.4	3,335.9	1,746.2	997.3	971.2 [10.5×]	–	–	–
*BigSeqKit*	10,168.6	5,264.5	2,711.5	1,412.2	814.6	545.4 [18.6×]	384.7	293.5	**234.9 [43.3×]**
**D_3_**									
*pyfastx*	13,075.3 [1.1×]	–	–	–	–	–	–	–	–
*seqkit*	14,281.6	8,161.7	5,009.6	3,184.1	1,832.4	1,054.9 [14.1×]	–	–	–
*BigSeqKit*	14,834.2	8,223.3	4,572.8	2,585.6	1,494.6	872.1 [17.0×]	532.8	365.9	**262.5 [56.5×]**
**D_4_**									
*pyfastx*	30,028.3 [1.05×]	–	–	–	–	–	–	–	–
*seqkit*	39,640.5	21,257.6	10,803.1	5,715.1	3,369.7	2,795.2 [11.3×]	–	–	–
*BigSeqKit*	31,615.2	16,832.1	8,531.9	4,433.3	2,466.8	1,609.9 [19.6×]	1,074.7	794.6	**633.5 [49.9×]**
**D_5_**									
*pyfastx*	27,876.5 [1.06×]	–	–	–	–	–	–	–	–
*seqkit*	31,301.8	16,884.7	9,141.1	4,698.4	2,971.8	2,802.9 [10.5×]	–	–	–
*BigSeqKit*	29,540.7	15,431.3	8,120.2	4,401.4	2,454.5	1,443.9 [20.5×]	908.1	599.5	**440.9 [67×]**
**D_6_**									
*pyfastx*	270,214 [1.02×]	–	–	–	–	–	–	–	–
*seqkit*	Out of Mem.	Out of Mem.	Out of Mem.	40,122	23,075	18,309 [15.0×]	–	–	–
*BigSeqKit*	275,680	141,095	72,110	37,140	19,810	11,477 [24.0×]	7,003	4,422	**3,080 [89.5×]**

**Table 5:** Execution times (seconds) using different number of cores: replace command. Highlighted are fastest time and number of times faster than sequential *BigSeqKit*

	1	2	4	8	16	32	64 (2 nodes)	128 (4 nodes)	256 (8 nodes)
**D_1_**									
*seqkit*	132.4 [1.02×]	–	–	–	–	–	–	–	–
*BigSeqKit*	134.5	69.5	36.1	25.0	18.7	12.7 [10.6×]	13.1	13.6	**12.5 [10.8×]**
**D_2_**									
*seqkit*	395.7 [1.04×]	–	–	–	–	–	–	–	–
*BigSeqKit*	410.6	213.5	110.1	74.5	56.9	29.7 [13.8×]	16.8	13.9	**13.5 [30.4×]**
**D_3_**									
*seqkit*	410.5 [0.99×]	–	–	–	–	–	–	–	–
*BigSeqKit*	406.7	209.5	109.4	74.0	56.1	29.5 [13.8×]	15.3	13.6	**12.9 [31.5×]**
**D_4_**									
*seqkit*	543.7 [1.05×]	–	–	–	–	–	–	–	–
*BigSeqKit*	570.3	293.5	109.4	74.0	55.1	29.4 [19.4×]	20.3	13.5	**12.5 [45.6×]**
**D_5_**									
*seqkit*	1,572.1 [1.03×]	–	–	–	–	–	–	–	–
*BigSeqKit*	1,621.7	819.9	420.1	217.2	115.1	62.9 [25.8×]	37.2	24.2	**18.5 [87.7×]**
**D_6_**									
*seqkit*	8,980.8 [1.07×]	–	–	–	–	–	–	–	–
*BigSeqKit*	9,620.8	5,000.3	2,605.2	1,364.2	717.7	387.5 [24.8×]	142.1	90.5	**60.2 [159.8×]**

**Table 6:** Execution times (seconds) using different number of cores: rmdup command. Highlighted are fastest time and number of times faster than sequential *BigSeqKit*

	1	2	4	8	16	32	64 (2 nodes)	128 (4 nodes)	256 (8 nodes)
**D_1_**									
*seqkit*	178.9 [1.01×]	–	–	–	–	–	–	–	–
*BigSeqKit*	180.5	94.3	50.2	35.1	27.1	15.8 [11.4×]	14.8	14.4	**13.8 [13.1×]**
**D_2_**									
*seqkit*	320.6 [1.04×]	–	–	–	–	–	–	–	–
*BigSeqKit*	333.3	174.7	93.5	65.9	49.9	26.5 [12.6×]	15.9	**14.1 [23.6×]**	15.0
**D_3_**									
*seqkit*	515.5 [0.91×]	–	–	–	–	–	–	–	–
*BigSeqKit*	469.5	246.7	182.7	127.5	96.1	51.4 [9.1×]	27.4	20.9	**20.6 [22.8×]**
**D_4_**									
*seqkit*	729.9 [0.99×]	–	–	–	–	–	–	–	–
*BigSeqKit*	720.5	378.5	197.5	139.7	102.9	54.0 [13.3×]	30.5	16.4	**14.1 [51.1×]**
**D_5_**									
*seqkit*	2,173.6 [0.97×]	–	–	–	–	–	–	–	–
*BigSeqKit*	2,100.2	1,110.4	612.3	341.2	195.1	115.2 [18.2×]	70.5	43.2	**28.1 [74.7×]**
**D_6_**									
*seqkit*	9,937.1 [1.11×]	–	–	–	–	–	–	–	–
*BigSeqKit*	11,022.3	5,578.5	3,006.7	1,709.6	1,004.1	600.1 [18.4×]	275.2	241.6	**228.8 [48.2×]**

**Table 7:** Execution times (seconds) using different number of cores: sample command. Highlighted are fastest time and number of times faster than sequential *BigSeqKit*

	1	2	4	8	16	32	64 (2 nodes)	128 (4 nodes)	256 (8 nodes)
**D_1_**									
*pyfastx*	308.2 [0.67×]	–	–	–	–	–	–	–	–
*seqkit*	196.1 [1.05×]	–	–	–	–	–	–	–	–
*BigSeqKit*	205.7	108.2	57.8	36.4	27.1	17.3 [11.9×]	15.1	15.4	**14.1 [14.6×]**
**D_2_**									
*pyfastx*	458.7 [1.12×]	–	–	–	–	–	–	–	–
*seqkit*	492.4 [1.04×]	–	–	–	–	–	–	–	–
*BigSeqKit*	514.5	271.7	143.8	98.1	76.1	42.2 [12.2×]	36.1	30.1	**26.4 [19.5×]**
**D_3_**									
*pyfastx*	450.2 [0.88×]	–	–	–	–	–	–	–	–
*seqkit*	491.7 [0.80×]	–	–	–	–	–	–	–	–
*BigSeqKit*	394.3	207.8	105.2	70.5	52.7	26.1 [15.1×]	22.1	19.2	**14.3 [27.6×]**
**D_4_**									
*pyfastx*	1,929.1 [0.99×]	–	–	–	–	–	–	–	–
*seqkit*	1,996.7 [0.96×]	–	–	–	–	–	–	–	–
*BigSeqKit*	1,912.8	1,000.5	529.3	365.8	283.4	156.3 [12.2×]	90.4	56.2	**36.5 [52.4×]**
**D_5_**									
*pyfastx*	1,567.7 [0.71×]	–	–	–	–	–	–	–	–
*seqkit*	1,057 [1.06×]	–	–	–	–	–	–	–	–
*BigSeqKit*	1,121.5	572.3	299.4	164.2	91.3	52.4 [21.4×]	33.6	25.1	**22.5 [49.8×]**
**D_6_**									
*pyfastx*	9,507.7 [1.16×]	–	–	–	–	–	–	–	–
*seqkit*	9,550 [1.16×]	–	–	–	–	–	–	–	–
*BigSeqKit*	11,070.2	5,539.5	2,812.3	1,543.6	876.2	515.9 [21.5×]	202	143.2	**109.5 [101.1×]**

**Table 8:** Execution times (seconds) using different number of cores: seq command. Highlighted are fastest time and number of times faster than sequential *BigSeqKit*

	1	2	4	8	16	32	64 (2 nodes)	128 (4 nodes)	256 (8 nodes)
**D_1_**									
*pyfastx*	151.8 [0.56×]	–	–	–	–	–	–	–	–
*seqkit*	234.4 [0.36×]	–	–	–	–	–	–	–	–
*BigSeqKit*	84.4	43.5	22.5	11.6	6.3	4.8 [17.6×]	4.7	3.7	**3.5 [24.1×]**
**D_2_**									
*pyfastx*	209.4 [1.15×]	–	–	–	–	–	–	–	–
*seqkit*	234.0 [1.03×]	–	–	–	–	–	–	–	–
*BigSeqKit*	240.9	128.5	65.0	34.6	19.5	10.7 [22.5×]	6.1	4.3	**4.0 [60.2×]**
**D_3_**									
*pyfastx*	400.5 [0.90×]	–	–	–	–	–	–	–	–
*seqkit*	541.2 [0.67×]	–	–	–	–	–	–	–	–
*BigSeqKit*	360.2	182.7	93.4	48.1	27.1	20.2 [17.8×]	8.6	**5.1 [65.5×]**	5.5
**D_4_**									
*pyfastx*	901.2 [1.13×]	–	–	–	–	–	–	–	–
*seqkit*	981.7 [1.03×]	–	–	–	–	–	–	–	–
*BigSeqKit*	1,014.7	508.8	257.1	129.1	66.3	36.6 [27.7×]	22.5	15.2	**10.6 [95.7×]**
**D_5_**									
*pyfastx*	1,051.4 [0.94×]	–	–	–	–	–	–	–	–
*seqkit*	1,165.5 [0.85×]	–	–	–	–	–	–	–	–
*BigSeqKit*	987.6	500.2	259.1	135.9	73.6	41.5 [23.8×]	26.1	17.9	**16.2 [60.9×]**
**D_6_**									
*pyfastx*	7,657.6 [1.23×]	–	–	–	–	–	–	–	–
*seqkit*	9,080.5 [1.04×]	–	–	–	–	–	–	–	–
*BigSeqKit*	9,420.3	4,712.1	2,400.3	1,323.4	755.5	430.3 [21.9×]	110.3	70.2	**55.5 [169.7×]**

**Table 9:** Execution times (seconds) using different number of cores: sort command. Highlighted are fastest time and number of times faster than sequential *BigSeqKit*

	1	2	4	8	16	32	64 (2 nodes)	128 (4 nodes)	256 (8 nodes)
**D_1_**									
*samtools*	1,590.3 [0.10×]	–	–	–	–	–	–	–	–
*seqkit*	169.0 [0.97×]	–	–	–	–	–	–	–	–
*BigSeqKit*	164.4	86.2	46.2	33.5	24.2	14.5 [11.3×]	13.8	13.5	**12.9 [12.7×]**
**D_2_**									
*samtools*	1,672.5 [0.25×]	–	–	–	–	–	–	–	–
*seqkit*	1,050.5 [0.40×]	–	–	–	–	–	–	–	–
*BigSeqKit*	422.8	221.6	117.6	81.7	62.1	34.9 [12.1×]	21.5	15.8	**13.2 [32.0×]**
**D_3_**									
*samtools*	1,203.5 [0.44×]	–	–	–	–	–	–	–	–
*seqkit*	497.5 [1.05×]	–	–	–	–	–	–	–	–
*BigSeqKit*	523.8	272.5	144.2	100.7	77.6	43.2 [12.1×]	26.5	18.6	**15.8 [33.1×]**
**D_4_**									
*samtools*	3,835.1 [0.36×]	–	–	–	–	–	–	–	–
*seqkit*	3,122.2 [0.44×]	–	–	–	–	–	–	–	–
*BigSeqKit*	1,377.3	708.5	372.5	243.7	171.5	94.6 [14.6×]	57.6	46.0	**36.0 [38.3×]**
**D_5_**									
*samtools*	1,899.6 [0.85×]	–	–	–	–	–	–	–	–
*seqkit*	3,350.4 [0.48×]	–	–	–	–	–	–	–	–
*BigSeqKit*	1,612.4	839.2	443.2	239.2	137.2	84.2 [19.1×]	53.4	40.2	**39.2 [41.1×]**
**D_6_**									
*samtools*	Out of Mem.	–	–	–	–	–	–	–	–
*seqkit*	Out of Mem.	–	–	–	–	–	–	–	–
*BigSeqKit*	18,309.6	9,439.6	4,899.2	2,592.8	1,444.4	839.7 [21.8×]	215.8	165.3	**139.6 [131.1×]**

Finally, we must highlight that one of the main reasons for the differences in the speedups between datasets running the same command with *BigSeqKit* is the load balance between threads. It will depend on the characteristics of the dataset: number of sequences and their length.

## Conclusions

Current state-of-the-art tools such as *seqkit, pyfastx*, and *samtools* are not ready for processing and manipulating very large FASTA and FASTQ files because all of them are mainly based on sequential processing. To that end, we have presented *BigSeqKit*, which parallelizes and optimizes the *seqkit* routines using the IgnisHPC computing framework. Since *seqkit* was programmed in Go, IgnisHPC was extended to support that language. As a consequence, IgnisHPC is nowadays the first parallel computing framework that supports Go. *BigSeqKit* can be easily installed on a local server or on a cluster. In addition, it can be used from the command line or as a library. Thanks to the multilanguage support of IgnisHPC, *BigSeqKit* routines can be called from C/C++, Python, Java, and Go codes.

Regarding the experimental results, *BigSeqKit* clearly outperforms *seqkit, pyfastx*, and *samtools* for all the tasks considered. On a single server, *BigSeqKit* is overall tens of times faster than those state-of-the-art tools, reaching speedups with respect to the *BigSeqKit* sequential time up to 27.7×. Considering an 8-node cluster, *BigSeqKit* is even faster, reaching speedups higher than 160×. It means that most of the tasks can be performed in just a few seconds. For instance, our toolkit effectively reduces the execution time of the locate command on our largest dataset from 3 days to a mere 0.8 hours. It is important to highlight that *seqkit* and *samtools* were unable to process that dataset with some routines due to memory issues, which confirms that current state-of-the-art tools are not well fitted for processing very large files.

As future work, we plan to add also the remainder *seqkit* commands not included in the current version of *BigSeqKit*: sliding, sana, fx2tab, tab2fx, convert, amplicon, fish, split, split2, restart, and mutate. Note that all of them are independent routines, so their implementation using IgnisHPC will be straightforward.

## Availability of Source Code and Requirements

Project name: BigSeqKit

- Project homepage: https://github.com/citiususc/BigSeqKit
- BiotoolsID: biotools:bigseqkit
- RRID: SCR_023592
- Operating system(s): Linux
- Programming language: Go
- Other requirements: IgnisHPC 2.2
- License: GNU GPL-3.0

## Supplementary Material

giad062_GIGA-D-23-00061_Original_Submission

giad062_GIGA-D-23-00061_Revision_1

giad062_GIGA-D-23-00061_Revision_2

giad062_GIGA-D-23-00061_Revision_3

giad062_Response_to_Reviewer_Comments_Original_Submission

giad062_Response_to_Reviewer_Comments_Revision_1

giad062_Response_to_Reviewer_Comments_Revision_2

giad062_Reviewer_1_Report_Original_Submission Umberto Ferraro Petrillo -- 4/11/2023 Reviewed

giad062_Reviewer_1_Report_Revision_1 Umberto Ferraro Petrillo -- 6/6/2023 Reviewed

giad062_Reviewer_2_Report_Original_Submission Weiguo Liu -- 4/12/2023 Reviewed

## Data Availability

The datasets supporting the results of this article are available as follows: *D*_1_ was obtained from the PacBio repository; *D*_2_, *D*_4_, and *D*_6_ from the International Genome Sample Resource (accession ids, SRR642648_1.filt, ERR4667750, and DRR002180_2) [27]; *D*_3_ from Ensembl [28] (assembly accession id, GCA_000001405.20); and *D*_5_ from UniProtKB—release 2022_03. All supporting data and materials are available in the *GigaScience* GigaDB database [29].

## Abbreviations

CLI: command-line interface; HPC: high-performance computing; JVM: Java Virtual Machine; NGS: next-generation sequencing; MPS: massive parallel sequencing.
