# Supplementary material for: BigSeqKit: a parallel Big Data toolkit to process FASTA and FASTQ files at scale
Source: Gigascience. 2023 Jul 31;12:giad062. doi: 10.1093/gigascience/giad062 (PMC10388699; doi:10.1093/gigascience/giad062)
Supplement: giad062_GIGA-D-23-00061_Revision_1 [file giad062_giga-d-23-00061_revision_1.pdf]

GigaScience

BigSeqKit: a parallel Big Data toolkit to process FASTA and FASTQ files at scale

--Manuscript Draft--

**Manuscript Number:**	GIGA-D-23-00061R1
**Full Title:**	BigSeqKit: a parallel Big Data toolkit to process FASTA and FASTQ files at scale
**Article Type:**	Technical Note
**Funding Information:**	Ministerio de Ciencia e Innovación (PLEC2021-007662) — Not applicable<br>Xunta de Galicia (ED431G/08) — Not applicable<br>Xunta de Galicia (ED431G-2019/04) — Not applicable<br>Xunta de Galicia (ED431C 2018/19) — Not applicable<br>Xunta de Galicia (ED431F 2020/08) — Not applicable<br>Horizon 2020 Framework Programme (HPC-EUROPA3 - INFRAIA-2016-1-730897) — Dr César Piñeiro<br>European Regional Development Fund — Not applicable
**Abstract:**	Background. High-throughput sequencing technologies have led to an unprecedented explosion in the amounts of sequencing data available, which are typically stored using FASTA and FASTQ files. We can find in the literature several tools to process and manipulate those type of files with the aim of transforming sequence data into biological knowledge. However, none of them are well fitted for processing efficiently very large files, likely in the order of terabytes in the following years, since they are based on sequential processing. Only some routines of the well-known seqkit tool are partly parallelized. In any case, its scalability is limited to use few threads on a single computing node.<br>Results. Our approach, BigSeqKit, takes advantage of an HPC-Big Data framework to parallelize and optimize the commands included in \emph{seqkit} with the aim of speeding up the manipulation of FASTA/FASTQ files. In this way, in most cases it is from tens to hundreds of times faster than several state-of-the-art tools. At the same time, our toolkit is easy to use and install on any kind of hardware platform (local server or cluster), and its routines can be used as a bioinformatics library or from the command line.<br>Conclusions. BigSeqKit is a very complete and ultra-fast toolkit to process and manipulate large FASTA and FASTQ files. It is publicly available at: https://github.com/citiususc/BigSeqKit
**Corresponding Author:**	Juan Carlos Pichel<br>Universidade de Santiago de Compostela<br>Santiago de Compostela, SPAIN
**Corresponding Author Secondary Information:**	
**Corresponding Author's Institution:**	Universidade de Santiago de Compostela
**Corresponding Author's Secondary Institution:**	
**First Author:**	César Piñeiro
**First Author Secondary Information:**	
**Order of Authors:**	César Piñeiro<br>Juan Carlos Pichel
**Order of Authors Secondary Information:**	
**Response to Reviewers:**	BigSeqKit: a parallel Big Data toolkit to process FASTA and FASTQ files at scale

Powered by Editorial Manager® and ProduXion Manager® from Aries Systems Corporation

César Piñeiro and Juan C. Pichel

Answers to the reviewers

We would like to thank the reviewers and editors for their insightful comments and suggestions about the paper. All the changes in the revised manuscript are highlighted in red. Detailed responses to reviewers are given below.

Reviewer #1: The manuscript addresses the problem of processing and manipulating large amounts of sequencing data stored in FASTA and FASTQ files. Based on the observation that most processing tools take a sequential approach, the authors present BigSeqKit, a parallelized and optimized toolkit that can be used on various hardware platforms and is tens to hundreds of times faster than other modern tools. It is described as a comprehensive and user-friendly toolkit for processing and manipulating large FASTA and FASTQ files. The paper also includes the results of experiments showing the superior performance of BigSeqKit compared to seqkit, its sequential counterpart, and other tools when a large number of processing kernels are used. Indeed, despite their very simple and inefficient structure, the FASTA and FASTQ file formats are still very common and will not be completely replaced by anything else in the foreseeable future. Against this background, the contribution of this paper might be of interest. However, I am not sure that the problem of speeding up traditional processing tools is as dramatic as the authors claim. A time saving of about 8 minutes for sorting the D3 dataset thanks to the use of 256 cores may not be so dramatic if the other steps of the analysis pipeline take hours or days, as can be the case for sequence alignments. That being said, I think the authors should provide a more solid justification for their contribution. This includes discussing, or at least anticipating, an application scenario where conventional tools fail in the first place and their approach is then needed.

****RESPONSE*****

We agree with the reviewer that it is important in the revised manuscript to better motivate/demonstrate why our approach is needed. With that goal in mind, we have extended our experimental evaluation including two larger datasets with the following characteristics (page 6):

• D5 (uniprot_trembl - FASTA - 104 GB): Number of sequences: 229.9M, Minimum length: 7, Average length: 351.6, Maximum length: 45.3K.
• D6 (DRR002180 2 - FASTQ - 395 GB): Number of sequences: 1.625B, Minimum length: 101, Average length: 101, Maximum length: 101.

In the original manuscript, D5 was only used with the faidx routine. Note that D6 is larger than the memory of one computing node (395 GB vs. 256 GB).

New performance results were added to Tables 3, 4, 5, 6, 7, 8 and 9, and the discussion about them is in pages 7, 8, 9 and 10 (changes highlighted in red in the revised manuscript). According to the new results, we prove our contribution taking into account the following arguments:

• pyfastx, samtools and seqkit take hours (and even days) to execute the different routines when considering the new datasets (see Tables 3-9). In this way, processing times are now significant in an analysis pipeline. For instance, the best sequential time of faidx, locate, replace, rmdup, sample, seq with D6 is about 2.1, 75.1, 2.5, 2.8, 2.6, hours, respectively. It means that, for example, the locate command requires more than 3 days of computation!

• seqkit and samtools were unable to process D6 with some routines (locate and sort) due to memory issues, which confirms that current state-of-the-art tools are not well fitted for processing very large files. In addition, it is expected that the size of the FASTA and FASTQ files increase even more in the near future. Note that BigSeqKit stores D6 compressed in memory when using one computing node since it exceeds the memory capacity of an individual server (see the Raw memory storage option in

Powered by Editorial Manager® and ProduXion Manager® from Aries Systems Corporation

the Background section -page 4).

• For all the commands considered and the new very large datasets, BigSeqKit is again the fastest tool. In addition, speedups are higher as data size grows, both considering 1, 2, 4 and 8 computing nodes. For instance, BigSeqKit is 169.7. faster than the sequential execution when considering D6 and the seq command (Table 8).

• BigSeqKit is able to reduce the time necessary to execute the locate command with our largest dataset D6 from 3 days to only 0.8 hours (Table 4). Therefore, the impact of using BigSeqKit is noticeable.

We have also modified the Conclusions (page 10) in the revised paper to include some of the results commented above. There is also a small change in the Intro (page 2). Links and IDs of the new datasets are provided in page 10 ("Availability of supporting data").

###################
###################

I have then some more punctual remarks:

-After a short review of existing FASTA/Q manipulation tools, the authors conclude that none of these tools is well fitted for the manipulation of large files of tens of GB. Why? As far as I can see, the same datasets used by the authors for their experiments are even larger than one hundred GB, however the authors have been able to process them using these tools.

****RESPONSE*****

This question is related to the previous one. To demonstrate the benefits of our approach we have included two larger datasets in our experimental evaluation for all the considered routines: D5 (104 GB) and D6 (395 GB). As we explained above, according to the results observed when processing both datasets, there are two main consequences that demonstrate that current state-of-the-art tools (pyfastx, samtools and seqkit) are not well fitted for very large files:

• There is a significant boost in the processing times when considering very large files. Now for all the commands studied, times range from 2 hours to more than 3 days. Therefore, the impact on the total time required by an analysis pipeline is very important. BigSeqKit is able to reduce those times noticeably. For example, pyfastx and seqkit require more than 2 hours to execute the sample command with D6, while BigSeqKit takes 109 seconds (see Table 7).

• If the dataset is big enough, there are memory issues that prevent samtools and seqkit to process the file when using several routines (locate and sort). These tools, and also pyfastx, are limited to store the data in the memory of a single node. BigSeqKit can use the memory of several nodes to split the data. In any case, even if there is only one computing node available, BigSeqKit can use additional storage options that allows it to process huge files larger than the memory of a node (see Background section in the manuscript -page 4):

– Raw memory: data is stored in a memory buffer using a serialized binary format. The buffer is compressed by Zlib.

– Disk: similar to raw memory but the buffer is stored as a POSIX file. Although the performance is significantly worse, it enables working with vast amounts of data that cannot be entirely kept in memory.

New performance results were added to Tables 3, 4, 5, 6, 7, 8 and 9, and the discussion about them is in pages 7, 8, 9 and 10 (changes highlighted in red in the revised manuscript).

###################
###################

Powered by Editorial Manager® and ProduXion Manager® from Aries Systems Corporation

-The paper gives the impression that BigSeqKit uses (at least) some of the code that imple.ments seqtk. However, it is unclear how this integration is done. Is seqtk executed as a child process in the BigSeqKit tasks, or has it been integrated at the source code or library level?

****RESPONSE*****

The reviewer is right in the sense that BigSeqKit reuses some parts of the seqkit code. However, BigSeqKit does not use seqkit as a child process or library. BigSeqKit is a reimplementation of seqkit functionalities that uses the IgnisHPC framework to deal with parallelism and performance. We analyzed the source code of seqkit and designed and implemented a new version of the commands that maintain the same behavior (and arguments) but operate in parallel. To do that we used the IgnisHPC API functions (see Background section). In addition, there are additional important changes explained in pages 4 and 5.

####################
####################

-The authors say that the use of IgnisHPC partitions makes it possible to improve seqtk in all operations where input data must be processed in multiple passes, since this data is held in memory. I expect this feature to be of great benefit when working with very large data sets. I would suggest the authors explicitly state in their experimental study which seqtk operations require multiple passes.

****RESPONSE*****

We did not use the multiple passes option in any of our experimental tests with seqkit. Note that this parameter reduces noticeably the performance of seqkit, so we have chosen not to use it to ensure a fair comparison with our tool. It is important to highlight that not all the seqkit commands support the ”two-pass” option. In our case, only sample and sort. For our new largest dataset D6, sample can still be executed without this parameter (see Table 7). On the other hand, the sort operation in seqkit cannot be executed with D6 due to memory issues even using the ”two-pass” argument.

Following the suggestion of the reviewer, the revised manuscript includes the fol.lowing sentence (page 6): ”Note that the ”two-pass” argument of seqkit was not used in the experiments.”

####################
####################

-To my surprise, no information was given about the overhead required to load the sequences to be processed into memory. In fact, some of the operations considered are I/O-bound and the resulting execution time is mainly due to the time required to read the sequences from disk to memory and vice versa. Is the load time included in the results reported by the authors?

****RESPONSE*****

Execution times for all the tools considered (BigSeqKit, seqkit, samtools and pyfastx) include the overhead of loading sequences into memory and the subsequent writing of results to disk.

Now the revised manuscript includes specifically that information (page 6).

####################
####################

In the IgnisHPC scenario, does each computational unit read a portion of the input files itself or are they loaded by a driver application and then distributed across the distributed system?

Powered by Editorial Manager® and ProduXion Manager® from Aries Systems Corporation

****RESPONSE*****

Each worker reads its portion of the input files, so the I/O operation is performed in parallel. There is one worker per computing node. Within each worker, its portion of the file is further divided among the available threads, improving the overall I/O performance.

Now the revised manuscript includes specifically that explanation (”Another implementation details” section -page 4).

###################
###################

In addition, the authors used an Infiniband-connected HPC infrastructure for their experiment. Do they use a remote storage server that exports a file system to all nodes of the distributed system? And, when using BigSeqKit to analyze very large files on all processing cores of a workstation, is there a potential performance/I/O bottleneck due to the controller’s limited bandwidth?

****RESPONSE*****

Our experiments were conducted using the Infiniband-connected HPC infrastructure at CESGA (Galicia Supercomputing Center, Spain). Within this infrastructure, a distributed Lustre file system is employed. It is a common practice to have dedicated storage nodes that handle the storage operations separately from the computational nodes. The Lustre system at CESGA is designed with data distribution and replication techniques to enhance performance and ensure data availability.

Now the revised manuscript explains that Lustre was used as distributed file system (page 6).

The reviewer is right that could be a potential bottleneck in the I/O performance due to the limited bandwidth of the memory controller when processing very large files. This could happen when executing commands with a very low ratio of operations per sequence. For example, the seq command. However, based on our experimental findings, that scenario is not happening since for all the commands and datasets considered, the scalability within a computing node is good.

Reviewer #2:

This paper provides a novel parallel toolkit named BigSeqKit to manipulate FASTA and FASTQ files. BigSeqKit takes advantage of the IgnisHPC to run on the distributed and local environment. And It takes advantage of the distributed performance of IgnisHPC to optimize various operations of seqkit, and provides some new functions. Moreover, it solves the data dependency problem of some commands in a distributed environment. BigSeqKit is tens to hundreds of times faster than several state-of-the-art tools. At the same time, BigSeqKit is easy to use and install on any kind of hardware platform (local server or cluster), and its routines can be used as a bioinformatics library or from the command line.

###################
###################

Questions:
1. In Figure 4. the locate operation is an independent operation according to the paper. But in D4, with 256 cores, why did it only achieve a 50x speedup?

****RESPONSE*****

The speedup is not higher due to a small fraction of the locate routine that should be executed sequentially. Amdahl’s law states that the overall speedup is limited by the proportion of the program or task that cannot be parallelized, even if the parallelizable

portion is improved significantly. In other words, the impact of optimizing a specific part of a system is limited by the non-parallelizable components. For instance, if only 1.5% of the code is sequential (that is, 98.5% executed in parallel), the theoretical maximum speedup achievable using 256 cores would be 53.. Note that this percentage varies depending of the dataset.
Now the revised manuscript includes this information in pages 6-7: ”Note that speedups of some routines are not higher when using 256 cores due to there is a small fraction of the code that should be executed sequentially (Amdahl's law)”.

####################
####################

2. Different data of the same type have very different speedups, for example, locate operation on dataset D1 and D3, can you explain why?

****RESPONSE*****

The differences in speedup between the locate operation on datasets D1 and D3 can be attributed to the characteristics of the datasets. D1 consists of 1.2 million sequences ranging from 85 to 19.7K in length, while D3 has only 639 sequences ranging from 970 to 248.9M in length (see page 6 in the revised manuscript). When processing both datasets in parallel, especially when the number of cores is high, it is difficult to find a good load balance between threads when the number of sequences is low and they are of very different size (up to 248.9M bases). That is the case of D3, and the reason why the speedups are different.

Now the revised paper includes this information in page 10: ”Finally, we must high.light that one of the main reasons for the differences in the speedups between datasets running the same command with BigSeqKit is the load balance between threads. It will depend on the characteristics of the dataset: number of sequences and their length.”.

####################
####################

3. How BigSeqKit ensure the integrity of the division data? For example, how to solve if a FASTQ sequence is divided into two partitions?

****RESPONSE*****

Each worker reads its portion of the input files, so the I/O operation is performed in parallel. There is one worker per computing node. Within each worker, its portion of the file is further divided among the available threads, improving the overall I/O performance.
In a text file, the separator is represented by '\n', while in FASTA and FASTQ files, it is '\n¿' and '\n@', respectively. If a thread begins reading its assigned portion and does not encounter the separator, it will ignore the entire input until the separator is found. Furthermore, if a thread has completed processing its portion, it will continue reading until the separator is encountered. This approach ensures a fully parallel and coordinated reading of the input file across multiple processes and threads, as specified by the user.

Now the revised manuscript includes specifically a summary of that explanation (”An.other implementation details” section -page 4).

####################
####################

4. In the conclusion section, the authors say: ”Considering an 8-nodes cluster, BigSeqKit is even faster, reaching speedups higher than 100.”, but only one data reaches speedup over 100x, why?

Following the suggestion of the first reviewer, we have we have extended our experimental evaluation including two larger datasets with the following characteristics (page 6):

	• D5 (uniprot_trembl - FASTA - 104 GB): Number of sequences: 229.9M, Minimum length: 7, Average length: 351.6, Maximum length: 45.3K.<br>• D6 (DRR002180 2 - FASTQ - 395 GB): Number of sequences: 1.625B, Minimum length: 101, Average length: 101, Maximum length: 101.<br><br>In the original manuscript, D5 was only used with the faidx routine. Note that D6 is larger than the memory of one computing node (395 GB vs. 256 GB).<br><br>As a result for all the commands considered and the new very large datasets, BigSeqKit is again the fastest tool. In addition, speedups are higher as data size grows, both considering 1, 2, 4 and 8 computing nodes. In particular, BigSeqKit is 144., 89.5., 159.8., 48.2., 101.1., 169.7. and 131.1. faster than the sequential execution when considering D6 and faidx, locate, replace, rmdup, sample, seq and sort commands, respectively. It means that in 5 of 7 routines achieves speedups higher than 100.<br><br>New performance results were added to Tables 3, 4, 5, 6, 7, 8 and 9, and the discussion about them is in pages 7, 8, 9 and 10 (changes highlighted in red in the revised manuscript). Conclusions (page 10) were also modified to reflect those results.<br><br>____<br><br>Editor:<br><br>In addition, please register any new software application in the bio.tools and SciCrunch.org databases to receive RRID (Research Resource Identification Initiative ID) and biotoolsID identifiers, and include these in your manuscript. Computational workflows should be regis.tered in workflowhub.eu and the DOIs cited in the relevant places in the manuscript. These will facilitate tracking, reproducibility and re-use of your tool.<br><br>****RESPONSE*****<br><br>Following the suggestion of the editor, BigSeqKit was registered in bio.tools and SciCrunh.org. Both IDs (and their corresponding links) were added to the repository information in the revised manuscript (page 10):<br>• BiotoolsID: biotools:bigseqkit<br>• RRID: SCR_023592
**Additional Information:**	
**Question**	**Response**
Are you submitting this manuscript to a special series or article collection?	No
**Experimental design and statistics**<br><br>Full details of the experimental design and statistical methods used should be given in the Methods section, as detailed in our Minimum Standards Reporting Checklist. Information essential to interpreting the data presented should be made available in the figure legends.<br><br>Have you included all the information requested in your manuscript?	Yes
**Resources**	Yes

A description of all resources used, including antibodies, cell lines, animals and software tools, with enough information to allow them to be uniquely identified, should be included in the Methods section. Authors are strongly encouraged to cite Research Resource Identifiers (RRIDs) for antibodies, model organisms and tools, where possible.<br><br>Have you included the information requested as detailed in our Minimum Standards Reporting Checklist?	
**Availability of data and materials**<br><br>All datasets and code on which the conclusions of the paper rely must be either included in your submission or deposited in publicly available repositories (where available and ethically appropriate), referencing such data using a unique identifier in the references and in the “Availability of Data and Materials” section of your manuscript.<br><br>Have you have met the above requirement as detailed in our Minimum Standards Reporting Checklist?	Yes

Powered by Editorial Manager® and ProduXion Manager® from Aries Systems Corporation

Manuscript

Click here to access/download;Manuscript;BigSeqKit_GigaScience_rev.pdf

OXFORD (GIGA)^n SCIENCE

GigaScience, 2017, 1–11

doi: xx.xxxx/xxxx
Manuscript in Preparation
Paper

PAPER

BigSeqKit: a parallel Big Data toolkit to process FASTA and FASTQ files at scale

César Piñeiro[1,*,†] and Juan C. Pichel[1,*]

[1]CiTIUS, Universidade de Santiago de Compostela, 15782 Santiago de Compostela, Spain

[*]cesaralfredo.pineiro@usc.es; juancarlos.pichel@usc.es
[†]Corresponding author

Abstract

Background. High-throughput sequencing technologies have led to an unprecedented explosion in the amounts of sequencing data available, which are typically stored using FASTA and FASTQ files. We can find in the literature several tools to process and manipulate those type of files with the aim of transforming sequence data into biological knowledge. However, none of them are well fitted for processing efficiently very large files, likely in the order of terabytes in the following years, since they are based on sequential processing. Only some routines of the well-known *seqkit* tool are partly parallelized. In any case, its scalability is limited to use few threads on a single computing node. **Results**. Our approach, *BigSeqKit*, takes advantage of an HPC-Big Data framework to parallelize and optimize the commands included in *seqkit* with the aim of speeding up the manipulation of FASTA/FASTQ files. In this way, in most cases it is from tens to hundreds of times faster than several state-of-the-art tools. At the same time, our toolkit is easy to use and install on any kind of hardware platform (local server or cluster), and its routines can be used as a bioinformatics library or from the command line. **Conclusions**. *BigSeqKit* is a very complete and ultra-fast toolkit to process and manipulate large FASTA and FASTQ files. It is publicly available at: https://github.com/citiususc/BigSeqKit.

Key words: FASTA/FASTQ files; Performance; Parallelism; Big Data

Introduction

The history of modern DNA sequencing started several decades ago, and since then, has seen astounding growth in sequencing capacity and speed. From the first genomes with a few thousand bases, DNA sequencing has advanced to sequence the human genome of 3 billion bases. In recent years, next-generation sequencing (NGS) technology, also known as massive parallel sequencing (MPS), has made it possible to expand the amount of sequencing data available. For example, the Illumina NovaSeq 6000[1] platform can generate a maximum output of 6 Tb of data and read about 20 billion sequences per run. Note that sequences, commonly named *reads*, are composed of ASCII characters representing a nucleotide (base) from the sequence. In the DNA case, we can only find four possible bases (A - adenine, C - cytosine, G - guanine and T - thymine).

1 https://www.illumina.com/systems/sequencing-platforms/novaseq.html [accessed 28 feb 2023]

The NGS raw data are mainly stored in FASTA [1] and FASTQ [2] text-based file formats. In particular, nucleotide and protein sequences are typically stored in the FASTA file format, whereas FASTQ is the most widely used format for sequencing read data. An example of FASTA file is shown in Figure 1. A sequence in FASTA format begins with a single-line description about the sequence in the subsequent lines. The description line is distinguished from the sequence data by a greater-than (>) symbol at the beginning. On the other hand, the FASTQ format was designed to handle the quality metrics of the sequences obtained from the sequencers. In FASTQ every four lines describe a sequence or read. An example is displayed in Figure 2. The information provided per read is: identifier and an optional description (first line), sequence (second line), and the quality score of the read (fourth line). An extra field, represented by symbol '+', is used as separator between the data and the quality information (third line).

Manipulating these files efficiently is essential to analyze and interpret data in any genomics pipeline. Common operations on FASTA and FASTQ files include searching, filtering, sampling, dedu-

Compiled on: May 25, 2023.
Draft manuscript prepared by the author.

```
>NG_008679.1:5001-38170 Homo sapiens paired box 6 (PAX6)
ACCCTCTTTTCTTATCATTGACATTTAAACTCTGGGGCAGGTCCTCGCGTAGAACGCGGCTGTCAGATCT
GCCACTTCCCCTGCCGAGCGGCGGTGAGAAGTGTGGGAACCGGCGCTGCCAGGCTCACCTGCCTCCCCGC
CCTCCGCTCCCAGGTAACCGCCCGGGCTCCGGCCCCGGCCCGGCTCGGGGCCCGCGGGGCCTCTCCGCTG
CCAGCGACTGCTGTCCCCAAATCAAAGCCCGCCCCAAGTGGCCCCGGGGCTTGATTTTTGCTTTTAAAAG
GAGGCATACAAAGATGGAAGCGAGTTACTGAGGGAGGGATAGGAAGGGGGGTGGAGGAGGGACTTGTCTT
TGCCGAGTGTGCTCTTCTGCAAAAGTAGCAAAATGTTCCACTCCTAAGAGTGGACTTCCAGTCCGGCCCT
GAGCTGGGAGTAGGGGGCGGGAGTCTGCTGCTGCTGTCTGCTAAAGCCACTCGCGACCGCGAAAAATGCA
GGAGGTGGGGACGCACTTTGCATCCAGACCTCCTCTGCATCGCAGTTCACGACATCCACGCTTGGGAAAG
TCCGTACCCGCGCCTGGAGCGCTTAAAGACACCCTGCCGCGGGTCGGGCGAGGTGCAGCAGAAGTTTCCC
GCGGTTGCAAAGTGCAGATGGCTGGACCGCAACAAAGTCTAGAGATGGGGTTCGTTTCTCAGAAAGACGC
```

Figure 1. Example of FASTA file showing the first part of the PAX6 gene (obtained from [3]).

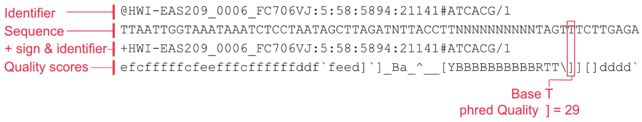

Figure 2. Example of FASTQ file format (obtained from [3]).

plication and sorting, among others. We can find several tools in the literature for FASTA/Q file manipulation such as *HTSeq* [4], *FASTX* [5], *fqtools* [6], *seqtk* [7], Biopython [8], samtools [9], *pyfadix* [10], *pyfastx* [11] and *seqkit* [12]. These tools can be classified according to how the sequences are parsed [11]. In the first category sequences are processed in order, which causes important overheads when extracting and randomly sampling sequences. That is the case of *HTSeq*, *FASTX*, *fqtools* and *seqtk*. In the second category we find tools that support random access to sequences by establishing an index file. Tools belonging to this category are more efficient in terms of performance and memory consumption. However, none of them are well fitted for processing very large files of hundreds of GB (likely TBs in the near future) since they are based on sequential processing. The exception is *seqkit* that allows some routines to use a few threads but, in any case, its scalability is very limited.

To deal with this issue, in this paper we introduce *BigSeqKit*[2], a parallel toolkit to manipulate FASTA and FASTQ files at scale with speed and scalability at its core. *BigSeqKit* takes advantage of IgnisHPC [13, 14], a computing engine that unifies the development, combination and execution of HPC and Big Data parallel tasks using different languages and programming models. As it was demonstrated, IgnisHPC outperforms the state-of-the-art framework Spark [15] in terms of performance and scalability running applications that represent the most typical algorithmic patterns in Big Data and scientific computing.

BigSeqKit uses the *seqkit* routines as basis since that toolkit covers a wide range of utilities and is one of the most used by the bioinformatics research community. As a consequence, *BigSeqKit* will offer the same functionalities and command interface[3] than *seqkit*. *BigSeqKit* can be used from the command line, but it is at the same time a library, so its routines can also be called from a C/C++, Python, Go or Java application.

Another important characteristic of *BigSeqKit* is that it is fully containerized, which isolates the execution environment from the physical system and avoids dependency problems. As a consequence, *BigSeqKit* is very easy to install and can run on a local server or on any type of cluster since it supports some of the most important resource and scheduler managers (e.g., Mesos [16], Nomad [17] and Slurm [18]).

Table 1. Some of the most important IgnisHPC API functions.

Type	Functions
Map	`map, flatmap, mapWithIndex, filter, keyBy, keys, values, mapPartitions, mapValues`, etc.
Reduce	`reduce, treeReduce, aggregate, treeAggregate, reduceByKey, aggregateByKey`, etc.
Group	`groupBy, groupByKey`
Sort	`sort, sortBy, sortByKey`
I/O	`parallelize,collect, top, take, saveAsObjectFile, saveAsTextFile, saveAsJsonFile`, etc
SQL	`union, join, distinct`
Math	`sample, sampleByKey, take, takeSample, count, countByKey, countByValue, max, min`, etc.
Balancing	`repartition, partitionByHash, partitionByRandom, partitionBy`
Persistence	`persist, cache, unpersist, uncache`

Background

IgnisHPC [13, 14] unifies the execution of Big Data and HPC workloads in the same computing engine. Unlike other frameworks such as Hadoop [19] and Spark [15], IgnisHPC has native support for multi-language applications using both JVM (Java Virtual Machine) and non-JVM-based languages. In this way, applications can be implemented using one or several programming languages following an API inspired by Spark's one.

The previous version of IgnisHPC supported natively C, C++, Java and Python. However, *seqkit* was implemented using the Go programming language. Since *BigSeqKit* parallelizes and optimizes the *seqkit* routines using IgnisHPC, it was necessary to add support for this language in the framework. Other solution would require to port the complete toolkit to a different language, which is a difficult and prone to errors task. It is worth noting that, to the best of our knowledge, nowadays IgnisHPC is the first parallel computing framework to include native support for this language. Considering Spark instead of IgnisHPC is not an option because, as it was demonstrated in [13], when using a non-native language code, data transfers between the JVM and external processes degrade noticeably the Spark's overall performance.

Go is a programming language with a simple syntax that was designed to be easy to learn and use. With the release of Go v1.18, the language included support for Generics, which allows the creation of functions, types, and methods that can work with any data type. This makes Go an effective and user-friendly way to implement Big Data interfaces. The implementation of Go in IgnisHPC is similar to that of C++, as both are compiled and statically typed languages. However, Go replaces the concept of inheritance with composition, which does not change the philosophy of use in IgnisHPC. Big Data functions are still accessible through the IgnisHPC API, and users can create their own code by implementing the same interfaces.

One of the key features of IgnisHPC is its use of containers to isolate and execute code. Containers are lightweight and portable, making it easy to run IgnisHPC on a variety of different clusters including both HPC (High-Performance Computing) and Big Data. IgnisHPC is also tolerant to failures, as the containers or processes can be easily restarted if there are issues. In particular, if some data is lost, IgnisHPC has enough information about how it was derived. In this way, only those operations needed to recompute the corresponding portion of data are performed.

We must highlight that although the IgnisHPC API[4] uses a se-

2 https://github.com/citiususc/BigSeqKit [accessed 28 feb 2023]
3 https://bioinf.shenwei.me/seqkit/usage [accessed 28 feb 2023]
4 https://ignishpc.readthedocs.io/en/latest/api.html [accessed 28 feb 2023]

quential notation, operations on data are performed in parallel. As we pointed out, the IgnisHPC API was inspired by the Spark API in such a way that IgnisHPC codes are easily understandable by users who are familiar with Spark. Table 1 shows a list of some of the most important functions supported by IgnisHPC. In particular:

- *Map functions:* The common characteristic to routines belonging to this type is that they apply the same function to each element in the data. As a result of the transformation, the output could be of different size with respect to the input.
- *Reduce functions:* `reduce` and `treeReduce` methods aggregate all the elements in the input data using a function. `aggregate` and `treeAggregate` are a sort of reduction where the type of the input and output data is different. In this case two functions are necessary, the first one is applied to each element in a data partition, and the second one combines the partial results obtained for each partition. `reduceByKey` and `aggregateByKey` are variations where the operation is performed only among elements with the same key in such a way that the final result is a set of unique pairs with values calculated using `reduce` or `aggregate` operations, respectively.
- *Group functions:* These methods group elements in a data frame according to their key value (`groupByKey`) or a user-defined function (`groupBy`).
- *Sort functions:* In order to sort elements, IgnisHPC provides three functions: `sort`, `sortByKey` and `sortBy`. The first method uses the natural order and does not need any additional function. `sortByKey` sorts the keys using their natural order. `sortBy` allows to use a user-defined function to specify the order of the elements. If the result of applying that function to two elements is *true*, then the first element should precede the second one. All methods support ascending and descending order.
- *SQL functions:* These functions operate on data frames. `union` concatenates two data frames, `join` merges elements of two data frames whose keys match, and `distinct` returns a new data frame after removing the duplicate records. These methods are necessary, for example, in many graph processing problems.
- *Other functions:* IgnisHPC implements several operations that return a value to the driver code, but they do not modify or generate new stored data. Spark refers to this type of operations as *actions*. For instance, IgnisHPC supports methods such as `count`, `take`, `takeSample` and `collect`. The most basic operation is `count` that returns the number of elements of a stored data collection. `collect` returns a collection with all the elements stored in the executors of a task. `take` applies a `collect` operation but obtains only the first *n* elements, where *n* is chosen by the user. `takeSample` returns a random sample of *n* elements from the distributed data, with or without replacement. Finally, another interesting routine is `parallelize`, which distributes the elements of a collection among the executors to form a distributed dataset. In this case new stored data is created.

It is worth noting that the IgnisHPC API functions allow users to parallelize a code with a high level of abstraction. In this way, it is only necessary to focus on data dependencies.

Methods

As we commented previously, *BigSeqKit* speeds up the *seqkit* routines through parallelization and optimization techniques. Table 2 shows the routines supported by the current version of *BigSeqKit*. Despite most of the commands in *seqkit* are sequential, we can classify each command implementation into three categories according to its inherent parallelism:

- Independent: it is a embarrassingly parallel workload. As a consequence, the computation could be applied to all sequences in parallel. An example is `seq`, a function that transforms sequences. In this case, the transformation only affects each sequence individually.
- Partially dependent: computations could be done in parallel, but the method requires some type of consensus to obtain the result. For instance, `stats` should merge the partial results computed for each sequence to calculate some statistics of the considered FASTA/Q file.
- Dependent: dependencies between sequences prevent the method from being executed in parallel. As a consequence, *BigSeqKit* requires a complete new algorithm to perform the same command in parallel. `rmdup` is a good example because with the aim of removing duplicated sequences it is necessary to read all of them before generating a result.

The integration, parallelization and optimization of each *seqkit* command in IgnisHPC will be different depending on its category. More details are provided below.

Independent routines

For these commands the computation can be applied to all sequences in parallel because there are no dependencies (communication) among them. In other words, routines belonging to this category can be processed using an embarrassingly parallel approach. Considering the IgnisHPC (and Spark) API, it is only necessary to use `map` functions to parallelize the computations. As we pointed out, the common characteristic to these API functions is that they apply the same operation to each element in the data.

The following *BigSeqKit* commands belong to this category: `seq`, `subseq`, `stats`, `fq2fa`, `fa2fq`, `translate`, `grep`, `locate`, `duplicate` and `replace` (see Table 2 for details).

Partially dependent routines

As we mentioned, this category includes commands in which computations can be done in parallel using `map` functions, but the methods require some type of consensus to get the desired outcome. This consensus can be easily implemented using the IgnisHPC API. The following *BigSeqKit* commands belong to this category:

- `stats`: statistics can be generated in parallel but the final result must be unique, so all partial results must be merged using a reduction (`reduce` operation in the IgnisHPC API).
- `head`: sequences should know their position inside the file to check if they are inside the head window. To do that, it is necessary to use `mapWithIndex`, a special `map` operation included in the IgnisHPC API that allows each element to know its global index within a data structure.
- `head-genome`: similar to `head`, but not all sequences are valid. In order to determine the window, invalid sequences must be removed first.
- `range`: also similar to `head`. Sequences should know their position inside the file to check if they are within the range window.
- `grep`: although this command was included in the previous category, a command option (`-delete-matched`) limits the number of results to just one per search pattern. In such cases, it is necessary to remove the extra results.
- `faidx`: also similar to `head`, sequences compute their offsets inside the input file using `mapPartitionWithIndex` and exchange the information between executors to perform a parallel indexing operation with a simple `map`.

Table 2. List of commands included in both *BigSeqKit* and *seqkit*. Those commands with an asterisk support new functionalities not included in *seqkit*.

**Basic commands**	
`seq`	Transform sequences (extract ID, filter by length, remove gaps, reverse complement, etc.)
`subseq`	Get subsequences by region/gtf/bed, including flanking sequences
`stats`	Simple statistics of FASTA/Q files: #seqs, min/max length, N50, Q20%, Q30%, etc.
`faidx`*	Create FASTA or FASTQ index file and extract subsequences
**Format conversion**	
`fa2fq`	Retrieve corresponding FASTQ records by a FASTA file
`fq2fa`	Convert FASTQ file to FASTA format
`translate`	Translate DNA/RNA to protein sequence
**Searching**	
`grep`	Search sequences by ID/name/sequence/sequence motifs
`locate`	Locate subsequences/motifs
**Set operations**	
`sample`	Sample sequences by number or proportion
`rmdup`	Remove duplicated sequences by ID/name/sequence
`common`	Find common sequences of multiple files by ID/name/sequence
`duplicate`	Duplicate sequences *N* times
`head`	Print first *N* FASTA/Q records
`head-genome`	Print sequences of the first genome with common prefixes in name
`pair`	Match up paired-end reads from two FASTQ files
`range`	Print FASTA/Q records in a range (start:end)
**Edit**	
`concat`	Concatenate sequences with the same ID from multiple files
`replace`	Replace name/sequence using a regular expression
`rename`	Rename duplicated IDs
**Ordering**	
`sort`	Sort sequences by ID/name/sequence/length
`shuffle`	Shuffle sequences

Dependent routines

Commands belonging to this category have an implementation in *seqkit* that by its nature cannot be parallelized. However, IgnisHPC allows us to define the implementation at a high level, which increases noticeably the productivity. Behaviors and functionalities will be preserved in *BigSeqKit* but through a complete new parallel implementation. In particular:

- `sample`: a sequential sampling can be performed in parallel if we split the sequences and run a sample for each partition. It was mathematically proven that sampling without replacement follows a hypergeometric function [20]. In this way, we can calculate the proportion of the sample that corresponds to each partition.
- `rmdup`: sequences are grouped (`groupBy` API function) using a hash with the ID, name or sequence. In those groups containing more than one element, a search for duplicates is carried out to remove them.
- `pair` and `concat`: sequences of the input files generate key-value pairs where the key is the ID and the value is the sequence with its index file (`map`). Pairs are unified by means of `union` and grouped using `groupByKey`. Afterwards, sequences in the same group are paired or concatenated if they belong to different files.
- `common`: the first stage of the command is the same one explained above for `pair` and `concat`. Then if a sequence can be found in all files, we check its index file, to avoid its deletion.
- `rename`: sequences are grouped (`groupBy`) using their ID, then IDs in the same group are renamed.
- `sort`: the sequential sort algorithm implemented in *seqkit* is replaced by a sample MergeSort [21] algorithm that can be efficiently executed in parallel in a distributed environment.
- `shuffle`: sequences shuffling can be implemented using the IgnisHPC API function `partitionByRandom`.

Another implementation details

In order to parallelize and integrate the *seqkit* routines into IgnisHPC it was necessary to start considering the sequence parser. It takes a stream of characters in FASTA and FASTQ format and generates a data structure with the sequence representation. In *seqkit*, this stream can be represented by a file or the standard input. In *BigSeqKit*, this stream is implemented using the IgnisHPC iterators, which grant the users access to the data partitions. In this way, *BigSeqKit* will read the data from a file and split it in multiple partitions, which facilitates their parallel processing. In particular, each worker reads a portion of the input file, so the I/O operation is performed in parallel. There is one worker per computing node. Within each worker, its portion of the file is further divided among the available threads, improving the overall I/O performance. As a result, the *seqkit* command arguments that affect file processing will have no effect in *BigSeqKit*. For example, the `-two-pass` option, which reads a file multiple times instead of storing all the sequences in memory, does not make sense in *BigSeqKit*. We must highlight that the fact of splitting the input files between several computing nodes in *BigSeqKit* means that the memory consumed by node is also split, which allows our tool to work with larger datasets. In addition, *BigSeqKit* also reduces the memory footprint by only storing the IDs and indices of each sequence.

Another important advantage of using IgnisHPC is how memory is handled. Users can choose a type of storage according to their particular case. For instance, if an input file is too large to be kept completely in the server memory, it could be stored compressed in memory or in disk. Performance would be lower, but it could be successfully processed. That scenario is not considered by *seqkit* that simply would raise an "out of memory" error. In particular, *BigSeqKit* supports the following storage options:

- *In-Memory:* it is the best performer since all data is stored in memory. It is the default option.
- *Raw memory:* data is stored in a memory buffer using a serialized

binary format. Extra memory consumption is minimal and the buffer is compressed by Zlib.
- *Disk:* similar to raw memory but the buffer is stored as a POSIX file. Although the performance is significantly worse, it enables working with vast amounts of data that cannot be entirely kept in memory.

On the other hand, `rmdup`, `common` and `pair` commands in *seqkit* use hash functions to check duplicates. It is well-known that hash functions can produce the same result for different values. This event is commonly known as a hash collision. However, *seqkit* does not check for collisions, so it is possible to generate incorrect results. *BigSeqKit* uses hashes to group sequences but then checks for collisions by comparing the real values.

Finally, *seqkit* and other state-of-the-art tools build index files (`faidx` routine) to speed up some other tasks (e.g., searches). Although *BigSeqKit* is also capable of creating those index files, it does not require them to improve its performance since data within IgnisHPC is already indexed. In other words, the index is created while reading the input file.

New functionalities

BigSeqKit not only enables the parallelization of *seqkit* functions, but also improves its algorithms to provide benefits even for sequential executions and includes additional functionalities. In particular, the `faidx` command in *seqkit* implements indexing of FASTA files using the *samtools* format, but FASTQ files are not supported. *BigSeqKit* adds support for this type of files and generates an index file using the *samtools* format as well. Note that this is the most widespread format and is also supported by other state-of-the-art tools. Therefore, *BigSeqKit* allows indexing of both FASTA and FASTQ files using the same syntax than *seqkit*.

How to use *BigSeqKit*

BigSeqKit can be used in two different ways. The first one is by means of a command-line interface (CLI). This approach is similar to the “command subcommand” structure adopted by *seqkit* [12]. In this way, it is only necessary to select a subcommand or routine (see a complete list in Table 2) and pass its arguments through command line. As we mentioned previously, to improve the usability and facilitate the adoption of *BigSeqKit*, it implements the same command interface than *seqkit*.

Since *BigSeqKit* runs within the IgnisHPC framework, it is necessary to send the *BigSeqKit* routine through the IgnisHPC submitter. For instance, if we are running *BigSeqKit* on a local server, the following expression uses the routine *seq* to print the name of the sequences included in a FASTA file to an output file:

```
ignis-submit ignishpc/full bigseqkit seq -n -o names.txt
     input-file.fa
```

Therefore, the syntax should be: `ignis-submit ignishpc/full bigseqkit <cmd> <arguments>`.

In addition, users can also specify through arguments the number of instances, cores and memory (in GB) to be used in the execution. By default, those values are set to 1. For example, we can execute the previous command using 2 cores:

```
ignis-submit ignishpc/full -p ignis.executor.cores=2
     bigseqkit seq -n -o names.txt input-file.fa
```

Unlike the other state-of-the-art tools, *BigSeqKit* can also be executed on a parallel cluster. Typical HPC clusters has Slurm [18] as the preferred resource manager, and Singularity [22] as container-based technology. In this case, users will send the job using the `ignis-slurm` submitter instead of `ignis-submit`.

```python
1  import ignis
2  import bigseqkit
3
4  # Initialization of the framework
5  ignis.Ignis.start()
6  # Resources/Configuration of the cluster
7  prop = ignis.IProperties()
8  prop["ignis.executor.image"] = "ignishpc/go"
9  prop["ignis.executor.instances"] = "1"
10 prop["ignis.executor.cores"] = "2"
11 prop["ignis.executor.memory"] = "1GB"
12 # Construction of the cluster
13 cluster = ignis.ICluster(prop)
14 # Initialization of a Go Worker
15 worker = ignis.IWorker(cluster, "go")
16 # Sequence reading
17 seqs = bigseqkit.readFASTA("file.fa", worker)
18 # Obtain Sequence names
19 names = bigseqkit.seq(seqs, name=True)
20 # Save the result
21 names.saveAsTextFile("names.txt")
22 # Stop the framework
23 ignis.Ignis.stop()
```

Figure 3. Example of Python code using the *BigSeqKit* routines.

On the other hand, *BigSeqKit* can also be used as a bioinformatics library. It is worth noting that *BigSeqKit* was implemented in Go language. However, thanks to the multi-language support provided by IgnisHPC, it is possible to call *BigSeqKit* routines from C/C++, Python, Java and Go applications without additional overhead. An example of Python code is shown in Figure 3. This example is equivalent to the previous one used in the explanation of the CLI. Since *BigSeqKit* has been created as a library, it only needs to be imported to be used. Functions in *BigSeqKit* do not use files as input, they use DataFrames instead, an abstract representation of parallel data used by IgnisHPC (similar to RDDs in Spark). Parameters are grouped in a data structure where each field represents the long names of a parameter. We must highlight that *BigSeqKit* functions can be linked (like system pipes using “|”), so the DataFrame generated by one can be used as input to another. In this way, integrate *BigSeqKit* routines in a more complex code is really easy.

The code starts initializing the IgnisHPC framework (line 5 in the figure). Next, a cluster of containers is configured and built (lines from 7 to 15). Multiple parameters can be used to configure the environment such as image, number of containers, number of cores and memory per container. In this example, we will use 1 node (instances) and 2 cores by node. After configuring the IgnisHPC execution environment, the *BigSeqKit* code actually starts. First, we read the input file (line 17). There is a different function for reading FASTA and FASTQ files. All the input sequences are stored as a single data structure. The next stage consists of printing the name of the sequences included in the FASTA file (line 19). The function takes as parameters the sequences and the options that specify its behavior. Finally, the names of the sequences are written to disk. It is important to highlight that lazy evaluation is performed, so functions are only executed when the result is required to be saved on disk.

Experimental Results

In this section we analyze the performance results obtained by *BigSeqKit* with respect to other state-of-the-art tools. In particular, we have considered *samtools*, *pyfastx* and *seqkit* for their perfor-

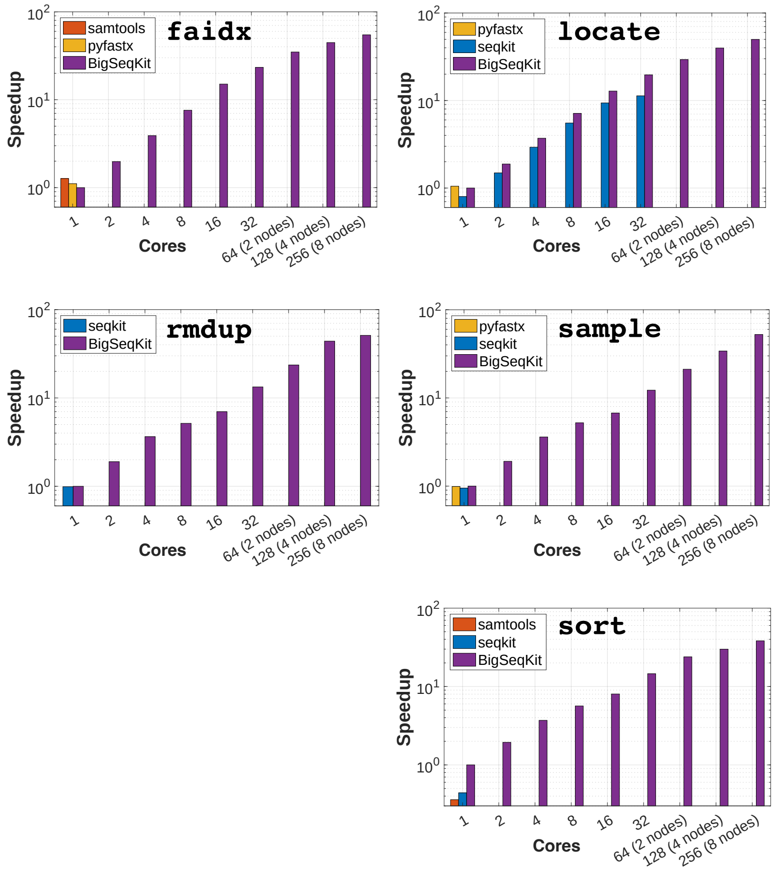

Figure 4. Speedups (in log scale) obtained by *BigSeqKit* and other state-of-the-art tools with respect to the *BigSeqKit* sequential time when executing different commands using D_4 as input. Note that `locate` was parallelized in *seqkit*.

mance and number of commands supported. Experiments were conducted using up to 8 computing nodes of the FinisTerrae III [5] supercomputer installed at CESGA (Spain). Each node contains a 32-core Intel Xeon Ice Lake 8352Y @2.2GHz processor and 256 GB of memory interconnected with Infiniband HDR 100. It is a Linux cluster running Rocky Linux v8.4 (kernel v4.18.0). We have used SingularityCE v3.9.7 (containers), IgnisHPC v2.2, *pyfastx* v0.8.4, *samtools* v1.16.1 and *seqkit* v2.3.1 (with Slurm as cluster manager and Lustre as distributed file system).

The performance evaluation was carried out using as input six different FASTA/FASTQ files that cover a wide variety of characteristics and sizes. The main features of these files are the following:

- D_1 (`m64013e_210227_222017.hifi_reads` - FASTA - 24 GB): Number of sequences: 1.2M, Minimum length: 85, Average length: 19.7K, Maximum length: 48.5K.
- D_2 (`SRR642648_1.filt` - FASTQ - 24.1 GB): Number of sequences: 98.7M, Minimum length: 100, Average length: 100, Maximum length: 100.
- D_3 (`Homo_sapiens.GRCh38.dna_sm.toplevel` - FASTA - 59.7 GB): Number of sequences: 639, Minimum length: 970, Average length: 98.8M, Maximum length: 248.9M.
- D_4 (`ERR4667750` - FASTQ - 79.1 GB): Number of sequences: 318.1M, Minimum length: 101, Average length: 101, Maximum length: 101.
- D_5 (`uniprot_trembl` - FASTA - 104 GB): Number of sequences: 229.9M, Minimum length: 7, Average length: 351.6, Maximum length: 45.3K.
- D_6 (`DRR002180_2` - FASTQ - 395 GB): Number of sequences: 1.625B, Minimum length: 101, Average length: 101, Maximum length: 101.

As example to illustrate the benefits of our tool, we will evaluate the following utilities (see Table 2 for a complete list of commands): `faidx` builds an index for FASTA/FASTQ files, `locate` locates sequences following some search pattern, `replace` replaces a name/sequence using a regular expression, `rmdup` removes duplicated sequences, `sample` selects sequences by number or proportion, `seq` transforms sequences (extract ID, filter by length, etc.) and removes gaps, and `sort` sorts sequences by ID/name/sequence/length. We will also include the performance results of the corresponding utilities, if exist, for *samtools*, *pyfastx* and *seqkit*. Execution times for all the tools considered include the overhead of loading sequences into memory and the subsequent writing of results to disk. Note that the "two-pass" argument of *seqkit* was not used in the experiments. Each result was computed as the median of five experiments. For the sake of reproducibility, all the codes and scripts used for performing the benchmarks are freely available at the *BigSeqKit* repository.

First, in order to provide an overall idea about the scalability and performance of *BigSeqKit* with respect to the other state-of-the-art tools, we will show the speedups obtained for the D_4 dataset using different number of cores. The behavior is very similar when considering the other datasets. Results in log scale are displayed in Figure 4. Speedups were calculated using as reference the sequential execution (1 core) of the corresponding *BigSeqKit* command. According to the results, several conclusions can be made. It can be observed that the scalability of *BigSeqKit* is quite good, reaching speedups up to 27.7× and 95.7× (`seq` command) using one server (32 cores) and eight computing nodes (256 cores), respectively. Note

5 https://www.cesga.es/en/infrastructures/computing/ [accessed 28 feb 2023]

Table 3. Execution times (seconds) using different number of cores: `faidx` command. Highlighted in blue, fastest time and number of times faster than sequential *BigSeqKit*.

	1	2	4	8	16	32	64 (2 nodes)	128 (4 nodes)	256 (8 nodes)
					D_1				
samtools	86.2 [1.03×]	–	–	–	–	–	–	–	–
pyfastx	109.2 [0.81×]	–	–	–	–	–	–	–	–
seqkit	75.4 [1.17×]	–	–	–	–	–	–	–	–
BigSeqKit	88.4	46.0	35.3	26.3	19.4	16.3 [5.4×]	13.6	12.3 [7.2×]	12.5
					D_2				
samtools	165.6 [1.06×]	–	–	–	–	–	–	–	–
pyfastx	177.9 [0.99×]	–	–	–	–	–	–	–	–
BigSeqKit	175.9	90.8	67.4	50.3	39.1	31.4 [5.6×]	23.4	19.1	15.5 [11.3×]
					D_3				
samtools	210.0 [0.77×]	–	–	–	–	–	–	–	–
pyfastx	131.2 [1.23×]	–	–	–	–	–	–	–	–
seqkit	131.8 [1.23×]	–	–	–	–	–	–	–	–
BigSeqKit	161.9	83.9	61.7	24.5	17.5	15.7 [10.3×]	13.6	13.4 [12.1×]	14.7
					D_4				
samtools	538.4 [1.27×]	–	–	–	–	–	–	–	–
pyfastx	615.5 [1.11×]	–	–	–	–	–	–	–	–
BigSeqKit	684.2	346.6	175.2	90.3	45.4	29.3 [23.3×]	19.6	15.3	12.5 [54.7×]
					D_5				
samtools	771.0 [1.08×]	–	–	–	–	–	–	–	–
pyfastx	634.3 [1.31×]	–	–	–	–	–	–	–	–
seqkit	1,096.2 [0.76×]	–	–	–	–	–	–	–	–
BigSeqKit	829.8	361.3	179.4	89.3	49.4	30.3 [27.4×]	23.6	19.3	16.5 [50.3×]
					D_6				
samtools	7,651.6 [1.14×]	–	–	–	–	–	–	–	–
pyfastx	7,712.5 [1.13×]	–	–	–	–	–	–	–	–
BigSeqKit	8,712.3	4,423.3	2,282.2	1,191.9	640.2	350.4 [24.9×]	129.5	85.3	60.5 [144×]

Table 4. Execution times (seconds) using different number of cores: `locate` command. Highlighted in blue, fastest time and number of times faster than sequential *BigSeqKit*.

	1	2	4	8	16	32	64 (2 nodes)	128 (4 nodes)	256 (8 nodes)
					D_1				
pyfastx	11,523.5 [1.0×]	–	–	–	–	–	–	–	–
seqkit	12,822.9	6,385.0	3,210.9	1,731.4	940.5	612.4 [18.8×]	–	–	–
BigSeqKit	11,486.2	6,286.1	3,180.0	1,637.3	850.9	470.6 [24.4×]	264.6	156.9	110.3 [104.1×]
					D_2				
pyfastx	8,841.2 [1.2×]	–	–	–	–	–	–	–	–
seqkit	12,319.8	6,909.4	3,335.9	1,746.2	997.3	971.2 [10.5×]	–	–	–
BigSeqKit	10,168.6	5264.5	2711.5	1412.2	814.6	545.4 [18.6×]	384.7	293.5	234.9 [43.3×]
					D_3				
pyfastx	13,075.3 [1.1×]	–	–	–	–	–	–	–	–
seqkit	14,281.6	8,161.7	5,009.6	3,184.1	1,832.4	1,054.9 [14.1×]	–	–	–
BigSeqKit	14,834.2	8,223.3	4,572.8	2,585.6	1,494.6	872.1 [17.0×]	532.8	365.9	262.5 [56.5×]
					D_4				
pyfastx	30,028.3 [1.05×]	–	–	–	–	–	–	–	–
seqkit	39,640.5	21,257.6	10,803.1	5,715.1	3,369.7	2,795.2 [11.3×]	–	–	–
BigSeqKit	31,615.2	16,832.1	8,531.9	4,433.3	2,466.8	1,609.9 [19.6×]	1,074.7	794.6	633.5 [49.9×]
					D_5				
pyfastx	27,876.5 [1.06×]	–	–	–	–	–	–	–	–
seqkit	31,301.8	16,884.7	9,141.1	4,698.4	2,971.8	2,802.9 [10.5×]	–	–	–
BigSeqKit	29,540.7	15,431.3	8,120.2	4,401.4	2,454.5	1,443.9 [20.5×]	908.1	599.5	440.9 [67×]
					D_6				
pyfastx	270,214 [1.02×]	–	–	–	–	–	–	–	–
seqkit	Out of Mem.	Out of Mem.	Out of Mem.	40,122	23,075	18,309 [15.0×]	–	–	–
BigSeqKit	275,680	141,095	72,110	37,140	19,810	11,477 [24.0×]	7,003	4,422	3,080 [89.5×]

that speedups of some routines are not higher when using 256 cores due to there is a small fraction of the code that should be executed sequentially (Amdahl's law).

While *samtools* and *pyfastx* routines are always processed sequentially, *seqkit* uses a multi-threaded approach to (partly) parallelize some commands. However, its scalability is limited to use a few threads on a single server (computing node). This is the case of `locate`. Its best speedup only reaches 11.3× (32 cores) while this value increases until 19.6× with *BigSeqKit*. If eight nodes are used, *BigSeqKit* is 49.9× faster than the sequential execution.

For all the commands studied, *BigSeqKit* clearly outperforms *samtools*, *pyfastx* and *seqkit*. There are only a few cases using one core where the speedups of these tools are slightly greater than 1. For instance, executing the `faidx` routine with *samtools* and *pyfastx*. However, other commands such as `sort` and `sample` are processed faster with *BigSeqKit* even using one core.

Tables from 3 to 9 display, for all the datasets, the execution times of *BigSeqKit* and the other state-of-the-art tools when running `faidx`, `locate`, `replace`, `rmdup`, `sample`, `seq` and `sort` utilities, respectively. Speedups with respect the sequential execution of the corresponding *BigSeqKit* command are shown between brackets. Highlighted in blue is shown the fastest time overall and the corresponding speedup. Note that *BigSeqKit* stores compressed in memory the largest dataset D_6 when using one computing node since it exceeds the memory capacity of an individual server (see the *Raw memory* storage option in the Background section).

For all the experiments conducted, *BigSeqKit* is always the fastest tool both considering a single server (one node) or several comput-

Table 5. Execution times (seconds) using different number of cores: `replace` command. Highlighted in blue, fastest time and number of times faster than sequential *BigSeqKit*.

	1	2	4	8	16	32	64 (2 nodes)	128 (4 nodes)	256 (8 nodes)
					D_1				
seqkit	132.4 [1.02×]	–	–	–	–	–	–	–	–
BigSeqKit	134.5	69.5	36.1	25.0	18.7	12.7 [10.6×]	13.1	13.6	**12.5 [10.8×]**
					D_2				
seqkit	395.7 [1.04×]	–	–	–	–	–	–	–	–
BigSeqKit	410.6	213.5	110.1	74.5	56.9	29.7 [13.8×]	16.8	13.9	**13.5 [30.4×]**
					D_3				
seqkit	410.5 [0.99×]	–	–	–	–	–	–	–	–
BigSeqKit	406.7	209.5	109.4	74.0	56.1	29.5 [13.8×]	15.3	13.6	**12.9 [31.5×]**
					D_4				
seqkit	543.7 [1.05×]	–	–	–	–	–	–	–	–
BigSeqKit	570.3	293.5	109.4	74.0	55.1	29.4 [19.4×]	20.3	13.5	**12.5 [45.6×]**
					D_5				
seqkit	1,572.1 [1.03×]	–	–	–	–	–	–	–	–
BigSeqKit	1,621.7	819.9	420.1	217.2	115.1	62.9[25.8×]	37.2	24.2	**18.5 [87.7×]**
					D_6				
seqkit	8,980.8 [1.07×]	–	–	–	–	–	–	–	–
BigSeqKit	9,620.8	5,000.3	2,605.2	1,364.2	717.7	387.5 [24.8×]	142.1	90.5	**60.2 [159.8×]**

Table 6. Execution times (seconds) using different number of cores: `rmdup` command. Highlighted in blue, fastest time and number of times faster than sequential *BigSeqKit*.

	1	2	4	8	16	32	64 (2 nodes)	128 (4 nodes)	256 (8 nodes)
					D_1				
seqkit	178.9 [1.01×]	–	–	–	–	–	–	–	–
BigSeqKit	180.5	94.3	50.2	35.1	27.1	15.8 [11.4×]	14.8	14.4	**13.8 [13.1×]**
					D_2				
seqkit	320.6 [1.04×]	–	–	–	–	–	–	–	–
BigSeqKit	333.3	174.7	93.5	65.9	49.9	26.5 [12.6×]	15.9	**14.1 [23.6×]**	15.0
					D_3				
seqkit	515.5 [0.91×]	–	–	–	–	–	–	–	–
BigSeqKit	469.5	246.7	182.7	127.5	96.1	51.4 [9.1×]	27.4	20.9	**20.6 [22.8×]**
					D_4				
seqkit	729.9 [0.99×]	–	–	–	–	–	–	–	–
BigSeqKit	720.5	378.5	197.5	139.7	102.9	54.0 [13.3×]	30.5	16.4	**14.1 [51.1×]**
					D_5				
seqkit	2,173.6 [0.97×]	–	–	–	–	–	–	–	–
BigSeqKit	2,100.2	1,110.4	612.3	341.2	195.1	115.2 [18.2×]	70.5	43.2	**28.1 [74.7×]**
					D_6				
seqkit	9,937.1 [1.11×]	–	–	–	–	–	–	–	–
BigSeqKit	11,022.3	5,578.5	3,006.7	1,709.6	1,004.1	600.1 [18.4×]	275.2	241.6	**228.8 [48.2×]**

Table 7. Execution times (seconds) using different number of cores: `sample` command. Highlighted in blue, fastest time and number of times faster than sequential *BigSeqKit*.

	1	2	4	8	16	32	64 (2 nodes)	128 (4 nodes)	256 (8 nodes)
					D_1				
pyfastx	308.2 [0.67×]	–	–	–	–	–	–	–	–
seqkit	196.1 [1.05×]	–	–	–	–	–	–	–	–
BigSeqKit	205.7	108.2	57.8	36.4	27.1	17.3 [11.9×]	15.1	15.4	**14.1 [14.6×]**
					D_2				
pyfastx	458.7 [1.12×]	–	–	–	–	–	–	–	–
seqkit	492.4 [1.04×]	–	–	–	–	–	–	–	–
BigSeqKit	514.5	271.7	143.8	98.1	76.1	42.2 [12.2×]	36.1	30.1	**26.4 [19.5×]**
					D_3				
pyfastx	450.2 [0.88×]	–	–	–	–	–	–	–	–
seqkit	491.7 [0.80×]	–	–	–	–	–	–	–	–
BigSeqKit	394.3	207.8	105.2	70.5	52.7	26.1 [15.1×]	22.1	19.2	**14.3 [27.6×]**
					D_4				
pyfastx	1,929.1 [0.99×]	–	–	–	–	–	–	–	–
seqkit	1,996.7 [0.96×]	–	–	–	–	–	–	–	–
BigSeqKit	1,912.8	1,000.5	529.3	365.8	283.4	156.3 [12.2×]	90.4	56.2	**36.5 [52.4×]**
					D_5				
pyfastx	1,567.7 [0.71×]	–	–	–	–	–	–	–	–
seqkit	1,057 [1.06×]	–	–	–	–	–	–	–	–
BigSeqKit	1,121.5	572.3	299.4	164.2	91.3	52.4 [21.4×]	33.6	25.1	**22.5 [49.8×]**
					D_6				
pyfastx	9,507.7 [1.16×]	–	–	–	–	–	–	–	–
seqkit	9,550 [1.16×]	–	–	–	–	–	–	–	–
BigSeqKit	11,070.2	5,539.5	2,812.3	1,543.6	876.2	515.9 [21.5×]	202	143.2	**109.5 [101.1×]**

ing nodes. In any case, let's take a look in detail of the behavior for each command:

- `faidx` (Table 3): *BigSeqKit* speedups range from 5.4× to 27.4× considering a single server (32 cores), and from 7.2× to 144× with 8 nodes. It means, for example, building the index file

Table 8. Execution times (seconds) using different number of cores: `seq` command. Highlighted in blue, fastest time and number of times faster than sequential *BigSeqKit*.

	1	2	4	8	16	32	64 (2 nodes)	128 (4 nodes)	256 (8 nodes)
					D_1				
pyfastx	151.8 [0.56×]	–	–	–	–	–	–	–	–
seqkit	234.4 [0.36×]	–	–	–	–	–	–	–	–
BigSeqKit	84.4	43.5	22.5	11.6	6.3	4.8 [17.6×]	4.7	3.7	**3.5 [24.1×]**
					D_2				
pyfastx	209.4 [1.15×]	–	–	–	–	–	–	–	–
seqkit	234.0 [1.03×]	–	–	–	–	–	–	–	–
BigSeqKit	240.9	128.5	65.0	34.6	19.5	10.7 [22.5×]	6.1	4.3	**4.0 [60.2×]**
					D_3				
pyfastx	400.5 [0.90×]	–	–	–	–	–	–	–	–
seqkit	541.2 [0.67×]	–	–	–	–	–	–	–	–
BigSeqKit	360.2	182.7	93.4	48.1	27.1	20.2 [17.8×]	8.6	**5.1 [65.5×]**	5.5
					D_4				
pyfastx	901.2 [1.13×]	–	–	–	–	–	–	–	–
seqkit	981.7 [1.03×]	–	–	–	–	–	–	–	–
BigSeqKit	1,014.7	508.8	257.1	129.1	66.3	36.6 [27.7×]	22.5	15.2	**10.6 [95.7×]**
					D_5				
pyfastx	1,051.4 [0.94×]	–	–	–	–	–	–	–	–
seqkit	1,165.5 [0.85×]	–	–	–	–	–	–	–	–
BigSeqKit	987.6	500.2	259.1	135.9	73.6	41.5 [23.8×]	26.1	17.9	**16.2 [60.9×]**
					D_6				
pyfastx	7,657.6 [1.23×]	–	–	–	–	–	–	–	–
seqkit	9,080.5 [1.04×]	–	–	–	–	–	–	–	–
BigSeqKit	9,420.3	4,712.1	2,400.3	1,323.4	755.5	430.3 [21.9×]	110.3	70.2	**55.5 [169.7×]**

Table 9. Execution times (seconds) using different number of cores: `sort` command. Highlighted in blue, fastest time and number of times faster than sequential *BigSeqKit*.

	1	2	4	8	16	32	64 (2 nodes)	128 (4 nodes)	256 (8 nodes)
					D_1				
samtools	1,590.3 [0.10×]	–	–	–	–	–	–	–	–
seqkit	169.0 [0.97×]	–	–	–	–	–	–	–	–
BigSeqKit	164.4	86.2	46.2	33.5	24.2	14.5 [11.3×]	13.8	13.5	**12.9 [12.7×]**
					D_2				
samtools	1,672.5 [0.25×]	–	–	–	–	–	–	–	–
seqkit	1,050.5 [0.40×]	–	–	–	–	–	–	–	–
BigSeqKit	422.8	221.6	117.6	81.7	62.1	34.9 [12.1×]	21.5	15.8	**13.2 [32.0×]**
					D_3				
samtools	1,203.5 [0.44×]	–	–	–	–	–	–	–	–
seqkit	497.5 [1.05×]	–	–	–	–	–	–	–	–
BigSeqKit	523.8	272.5	144.2	100.7	77.6	43.2 [12.1×]	26.5	18.6	**15.8 [33.1×]**
					D_4				
samtools	3,835.1 [0.36×]	–	–	–	–	–	–	–	–
seqkit	3,122.2 [0.44×]	–	–	–	–	–	–	–	–
BigSeqKit	1,377.3	708.5	372.5	243.7	171.5	94.6 [14.6×]	57.6	46.0	**36.0 [38.3×]**
					D_5				
samtools	1,899.6 [0.85×]	–	–	–	–	–	–	–	–
seqkit	3,350.4 [0.48×]	–	–	–	–	–	–	–	–
BigSeqKit	1,612.4	839.2	443.2	239.2	137.2	84.2 [19.1×]	53.4	40.2	**39.2 [41.1×]**
					D_6				
samtools	Out of Mem.	–	–	–	–	–	–	–	–
seqkit	Out of Mem.	–	–	–	–	–	–	–	–
BigSeqKit	18,309.6	9,439.6	4,899.2	2,592.8	1,444.4	839.7 [21.8×]	215.8	165.3	**139.6 [131.1×]**

for our largest dataset D_6 (395 GB) in just 5.8 minutes (single server), while *samtools* and *pyfastx* require about 2.1 hours. This time decreases to one minute when *BigSeqKit* uses 8 nodes. As mentioned previously, the `faidx` routine in *seqkit* does not support FASTQ files (D_2, D_4 and D_6).

- `locate` (Table 4): the searching routines, `grep` and `locate`, are very expensive in terms of computations. Note that considering sequential processing, `locate` takes more than 3 hours to process our smallest dataset D_1 independently of the tool considered. This time increases to more than 3 days of computation for D_6. *seqkit* has a multi-thread version of `locate`, which obtains speedups from 10.5× to 18.8×. These speedups are always lower to the ones obtained by *BigSeqKit* on a single server. It is important to highlight that *seqkit* raises an out of memory error when processing D_6 with 1, 2 and 4 cores. On the other hand, when using 8 nodes, *BigSeqKit* achieves noticeable speedups up to 104.1×. In this way, it is able to reduce the time necessary to execute the `locate` command with our largest dataset D_6 from 3 days to only 0.8 hours.
- `replace` (Table 5): this routine (or an equivalent) is not supported by *samtools* and *pyfastx*. In this case, *BigSeqKit* is from tens to hundreds of times faster than *seqkit*, reaching speedups up to 159.8×.
- `rmdup` (Table 6): this routine is also not supported by *samtools* and *pyfastx*. In this case, *BigSeqKit* is tens of times faster than *seqkit*, achieving a maximum speedup of 74.7× when removing the duplicated sequences in D_5.
- `sample` (Table 7): operation not supported by *samtools*. *BigSeqKit* is again faster than the other tools, increasing the speedups as the input datasize grows. It can be observed that *BigSeqKit* is able to sample sequences in seconds. For instance, *pyfastx* and *seqkit* take about 3 hours to process D_6, while *BigSeqKit* requires just 2 minutes.
- `seq` (Table 8): operation not supported by *samtools*. Performance results are similar to the `sample` ones in such a way that *BigSeqKit* filters sequences by ID in few seconds, achieving a noticeable

speedup of 169.7×. It should be noted that among the routines examined in this study, `seq` is the least computationally demanding.

- `sort` (Table 9): this routine was not included in *pyfastx*. In general, the performance of *samtools* and *seqkit* is poor. And, most importantly, both tools produce memory errors when processing the largest dataset D_6, so it cannot be sorted. However, *BigSeqKit* sorts D_6 21.8× and 131.1× faster than the sequential version using a single server and 8 computing nodes, respectively. It means that the time decreases from 5 hours to barely 2 minutes.

Finally, we must highlight that one of the main reasons for the differences in the speedups between datasets running the same command with *BigSeqKit* is the load balance between threads. It will depend on the characteristics of the dataset: number of sequences and their length.

Conclusions

Current state-of-the-art tools such as *seqkit*, *pyfastx* and *samtools* are not ready for processing and manipulating very large FASTA and FASTQ files because all of them are mainly based on sequential processing. To that end, we have presented *BigSeqKit*, which parallelizes and optimizes the *seqkit* routines using the IgnisHPC computing framework. Since *seqkit* was programmed in Go, IgnisHPC was extended to support that language. As a consequence, IgnisHPC is nowadays the first parallel computing framework that supports Go. *BigSeqKit* can be easily installed on a local server or on a cluster. In addition, it can be used from the command line or as a library. Thanks to the multi-language support of IgnisHPC, *BigSeqKit* routines can be called from C/C++, Python, Java and Go codes.

Regarding the experimental results, *BigSeqKit* clearly outperforms *seqkit*, *pyfastx* and *samtools* for all the tasks considered. On a single server, *BigSeqKit* is overall tens of times faster than those state-of-the-art tools, reaching speedups with respect to the *BigSeqKit* sequential time up to 27.7×. Considering an 8-nodes cluster, *BigSeqKit* is even faster, reaching speedups higher than 160×. It means that most of the tasks can be performed in just a few seconds. For instance, our toolkit effectively reduces the execution time of the `locate` command on our largest dataset from 3 days to a mere 0.8 hours. It is important to highlight that *seqkit* and *samtools* were unable to process that dataset with some routines due to memory issues, which confirms that current state-of-the-art tools are not well fitted for processing very large files.

As future work we plan to add also the remainder *seqkit* commands not included in the current version of *BigSeqKit*: `sliding`, `sana`, `fx2tab`, `tab2fx`, `convert`, `amplicon`, `fish`, `split`, `split2`, `restart` and `mutate`. Note that all of them are independent routines, so their implementation using IgnisHPC will be straightforward.

Availability of source code and requirements

- Project name: BigSeqKit
- Project home page: https://github.com/citiususc/BigSeqKit
- BiotoolsID: biotools:bigseqkit
- RRID: SCR_023592
- Operating system(s): Linux
- Programming language: Go
- Other requirements: IgnisHPC 2.2
- License: GNU GPL-3.0

Availability of supporting data and materials

The datasets supporting the results of this article are available in: D_1 was obtained from the PacBio repository, D_2, D_4 and D_6 from the International Genome Sample Resource (accession ids, SRR642648_1.filt, ERR4667750 and DRR002180_2) [23], D_3 from Ensembl [24] (assembly accession id, GCA_000001405.20), and D_5 from UniProtKB - release 2022_03.

Declarations

List of abbreviations

(CLI) Command-Line Interface, (HPC) High-Performance Computing, (JVM) Java Virtual Machine, (NGS) Next-Generation Sequencing, (MPS) Massive Parallel Sequencing.

Ethical Approval

Not applicable.

Consent for publication

Not applicable.

Competing Interests

The authors declare that they have no competing interests.

Funding

This work was supported by MICINN, Spain [PLEC2021-007662]; Xunta de Galicia, Spain [ED431G/08, ED431G-2019/04, ED431C 2018/19, and ED431F 2020/08]; European Commission RIA - H2020 [HPC-EUROPA3 - INFRAIA-2016-1-730897] and European Regional Development Fund (ERDF).

Author's Contributions

César Piñeiro: Methodology, Software Development, Conducted Experiments, and Contributed to Writing.
Juan C. Pichel: Conceptualization, Methodology, Supervision, Writing and Revision.

Acknowledgements

C. Piñeiro acknowledges the support of Marco Aldinucci (University of Torino, Italy) and the computer resources provided by CINECA (Italy) used in the preliminary tests. Authors also wish to thank CESGA (Galicia, Spain) for providing access to their supercomputing facilities.

References

1. Pearson WR, Lipman DJ. Improved tools for biological sequence comparison. Proceedings of the National Academy of Sciences 1988;85(8):2444–2448.
2. Cock PJ, Fields CJ, Goto N, Heuer ML, Rice PM. The Sanger FASTQ file format for sequences with quality scores, and the Solexa/Illumina FASTQ variants. Nucleic acids research 2010;38(6):1767–1771.
3. Akalin A. Computational Genomics with R. Chapman and Hall/CRC; 2020.
4. Anders S, Pyl PT, Huber W. HTSeq — a Python framework to work with high-throughput sequencing data. Bioinformatics 2014 09;31(2):166–169.

5. Gordon A, Hannon G, FASTX-Toolkit: FASTQ/A short-reads pre-processing tools; 2010. http://hannonlab.cshl.edu/fastx_toolkit [accessed 28 feb 2023].
6. Droop AP. fqtools: an efficient software suite for modern FASTQ file manipulation. Bioinformatics 2016 02;32(12):1883–1884.
7. Li H, seqtk: toolkit for processing sequences in FASTA/Q formats; 2012. https://github.com/lh3/seqtk [accessed 28 feb 2023].
8. Cock PJ, Antao T, Chang JT, Chapman BA, Cox CJ, Dalke A, et al. Biopython: freely available Python tools for computational molecular biology and bioinformatics. Bioinformatics 2009;25(11):1422–1423.
9. Li H, Handsaker B, Wysoker A, Fennell T, Ruan J, Homer N, et al. The sequence alignment/map format and SAMtools. Bioinformatics 2009;25(16):2078–2079.
10. Shirley MD, Ma Z, Pedersen BS, Wheelan SJ. Efficient "pythonic" access to FASTA files using pyfaidx. PeerJ PrePrints; 2015.
11. Du L, Liu Q, Fan Z, Tang J, Zhang X, Price M, et al. Pyfastx: a robust Python package for fast random access to sequences from plain and gzipped FASTA/Q files. Briefings in Bioinformatics 2020 12;22(4). https://github.com/lmdu/pyfastx [accessed 28 feb 2023].
12. Shen W, Le S, Li Y, Hu F. SeqKit: a cross-platform and ultrafast toolkit for FASTA/Q file manipulation. PloS One 2016;11(10):e0163962.
13. Piñeiro C, Martínez-Castaño R, Pichel JC. Ignis: An efficient and scalable multi-language Big Data framework. Future Generation Computer Systems 2020;105:705–716.
14. Piñeiro C, Pichel JC. A unified framework to improve the interoperability between HPC and Big Data languages and programming models. Future Generation Computer Systems 2022;134:123–139.
15. Zaharia M, Chowdhury M, Franklin MJ, Shenker S, Stoica I. Spark: Cluster Computing with Working Sets. In: HotCloud; 2010. p. 10–10.
16. Hindman B, Konwinski A, Zaharia M, Ghodsi A, Joseph AD, Katz R, et al. Mesos: A Platform for Fine-Grained Resource Sharing in the Data Center. In: Proc. of the 8th USENIX Conf. on Networked Systems Design and Implementation; 2011. p. 295–308.
17. HashiCorp, Nomad: workload orchestration made easy;. [Online; accessed 28 feb 2023]. https://www.nomadproject.io.
18. Yoo AB, Jette MA, Grondona M. Slurm: Simple linux utility for resource management. In: Workshop on job scheduling strategies for parallel processing; 2003. p. 44–60.
19. White T. Hadoop: The Definitive Guide. 4th ed. O'Reilly Media, Inc.; 2015.
20. Sanders P, Lamm S, Hübschle-Schneider L, Schrade E, Dachsbacher C. Efficient parallel random sampling—vectorized, cache-efficient, and online. ACM Transactions on Mathematical Software (TOMS) 2018;44(3):1–14.
21. Li X, Lu P, Schaeffer J, Shillington J, Wong PS, Shi H. On the Versatility of Parallel Sorting by Regular Sampling. Parallel Computing 1993;19:1079–1103.
22. Kurtzer GM, Sochat V, Bauer MW. Singularity: Scientific containers for mobility of compute. PLOS ONE 2017 05;12(5):1–20.
23. Fairley S, Lowy-Gallego E, Perry E, Flicek P. The International Genome Sample Resource (IGSR) collection of open human genomic variation resources. Nucleic Acids Research 2019 10;48(D1):D941–D947.
24. Cunningham F, Allen JE, Allen J, Alvarez-Jarreta J, Amode M, Armean I, et al. Ensembl 2022. Nucleic Acids Research 2021 11;50(D1):D988–D995.
