# Supplementary material for: BigSeqKit: a parallel Big Data toolkit to process FASTA and FASTQ files at scale
Source: Gigascience. 2023 Jul 31;12:giad062. doi: 10.1093/gigascience/giad062 (PMC10388699; doi:10.1093/gigascience/giad062)
Supplement: giad062_Response_to_Reviewer_Comments_Original_Submission [file giad062_response_to_reviewer_comments_original_submission.pdf]

Author's Response To Reviewer Comments

Close

BigSeqKit: a parallel Big Data toolkit to process FASTA and FASTQ files at scale
César Piñeiro and Juan C. Pichel

Answers to the reviewers

We would like to thank the reviewers and editors for their insightful comments and suggestions about the paper. All the changes in the revised manuscript are highlighted in red. Detailed responses to reviewers are given below.

——————————————————————————————–

Reviewer #1: The manuscript addresses the problem of processing and manipulating large amounts of sequencing data stored in FASTA and FASTQ files. Based on the observation that most processing tools take a sequential approach, the authors present BigSeqKit, a parallelized and optimized toolkit that can be used on various hardware platforms and is tens to hundreds of times faster than other modern tools. It is described as a comprehensive and user-friendly toolkit for processing and manipulating large FASTA and FASTQ files. The paper also includes the results of experiments showing the superior performance of BigSeqKit compared to seqkit, its sequential counterpart, and other tools when a large number of processing kernels are used. Indeed, despite their very simple and inefficient structure, the FASTA and FASTQ file formats are still very common and will not be completely replaced by anything else in the foreseeable future. Against this background, the contribution of this paper might be of interest. However, I am not sure that the problem of speeding up traditional processing tools is as dramatic as the authors claim. A time saving of about 8 minutes for sorting the D3 dataset thanks to the use of 256 cores may not be so dramatic if the other steps of the analysis pipeline take hours or days, as can be the case for sequence alignments. That being said, I think the authors should provide a more solid justification for their contribution. This includes discussing, or at least anticipating, an application scenario where conventional tools fail in the first place and their approach is then needed.

****RESPONSE*****

We agree with the reviewer that it is important in the revised manuscript to better motivate/demonstrate why our approach is needed. With that goal in mind, we have extended our experimental evaluation including two larger datasets with the following characteristics (page 6):

• D5 (uniprot_trembl - FASTA - 104 GB): Number of sequences: 229.9M, Minimum length: 7, Average length: 351.6, Maximum length: 45.3K.
• D6 (DRR002180 2 - FASTQ - 395 GB): Number of sequences: 1.625B, Minimum length: 101, Average length: 101, Maximum length: 101.

In the original manuscript, D5 was only used with the faidx routine. Note that D6 is larger than the memory of one computing node (395 GB vs. 256 GB).

New performance results were added to Tables 3, 4, 5, 6, 7, 8 and 9, and the discussion about them is in pages 7, 8, 9 and 10 (changes highlighted in red in the revised manuscript). According to the new results, we prove our contribution taking into account the following arguments:

• pyfastx, samtools and seqkit take hours (and even days) to execute the different routines when considering the new datasets (see Tables 3-9). In this way, processing times are now significant in an analysis pipeline. For instance, the best sequential time of faidx, locate, replace, rmdup, sample, seq with

D6 is about 2.1, 75.1, 2.5, 2.8, 2.6, hours, respectively. It means that, for example, the locate command requires more than 3 days of computation!

• seqkit and samtools were unable to process D6 with some routines (locate and sort) due to memory issues, which confirms that current state-of-the-art tools are not well fitted for processing very large files. In addition, it is expected that the size of the FASTA and FASTQ files increase even more in the near future. Note that BigSeqKit stores D6 compressed in memory when using one computing node since it exceeds the memory capacity of an individual server (see the Raw memory storage option in the Background section -page 4).

• For all the commands considered and the new very large datasets, BigSeqKit is again the fastest tool. In addition, speedups are higher as data size grows, both considering 1, 2, 4 and 8 computing nodes. For instance, BigSeqKit is 169.7. faster than the sequential execution when considering D6 and the seq command (Table 8).

• BigSeqKit is able to reduce the time necessary to execute the locate command with our largest dataset D6 from 3 days to only 0.8 hours (Table 4). Therefore, the impact of using BigSeqKit is noticeable.

We have also modified the Conclusions (page 10) in the revised paper to include some of the results commented above. There is also a small change in the Intro (page 2). Links and IDs of the new datasets are provided in page 10 ("Availability of supporting data").

###################
###################

I have then some more punctual remarks:

-After a short review of existing FASTA/Q manipulation tools, the authors conclude that none of these tools is well fitted for the manipulation of large files of tens of GB. Why? As far as I can see, the same datasets used by the authors for their experiments are even larger than one hundred GB, however the authors have been able to process them using these tools.

****RESPONSE*****

This question is related to the previous one. To demonstrate the benefits of our approach we have included two larger datasets in our experimental evaluation for all the considered routines: D5 (104 GB) and D6 (395 GB). As we explained above, according to the results observed when processing both datasets, there are two main consequences that demonstrate that current state-of-the-art tools (pyfastx, samtools and seqkit) are not well fitted for very large files:

• There is a significant boost in the processing times when considering very large files. Now for all the commands studied, times range from 2 hours to more than 3 days. Therefore, the impact on the total time required by an analysis pipeline is very important. BigSeqKit is able to reduce those times noticeably. For example, pyfastx and seqkit require more than 2 hours to execute the sample command with D6, while BigSeqKit takes 109 seconds (see Table 7).

• If the dataset is big enough, there are memory issues that prevent samtools and seqkit to process the file when using several routines (locate and sort). These tools, and also pyfastx, are limited to store the data in the memory of a single node. BigSeqKit can use the memory of several nodes to split the data. In any case, even if there is only one computing node available, BigSeqKit can use additional storage options that allows it to process huge files larger than the memory of a node (see Background section in the manuscript -page 4):

– Raw memory: data is stored in a memory buffer using a serialized binary format. The buffer is compressed by Zlib.

– Disk: similar to raw memory but the buffer is stored as a POSIX file. Although the performance is

significantly worse, it enables working with vast amounts of data that cannot be entirely kept in memory.

New performance results were added to Tables 3, 4, 5, 6, 7, 8 and 9, and the discussion about them is in pages 7, 8, 9 and 10 (changes highlighted in red in the revised manuscript).

###################
###################

-The paper gives the impression that BigSeqKit uses (at least) some of the code that imple.ments seqtk. However, it is unclear how this integration is done. Is seqtk executed as a child process in the BigSeqKit tasks, or has it been integrated at the source code or library level?

****RESPONSE*****

The reviewer is right in the sense that BigSeqKit reuses some parts of the seqkit code. However, BigSeqKit does not use seqkit as a child process or library. BigSeqKit is a reimplementation of seqkit functionalities that uses the IgnisHPC framework to deal with parallelism and performance. We analyzed the source code of seqkit and designed and implemented a new version of the commands that maintain the same behavior (and arguments) but operate in parallel. To do that we used the IgnisHPC API functions (see Background section). In addition, there are additional important changes explained in pages 4 and 5.

###################
###################

-The authors say that the use of IgnisHPC partitions makes it possible to improve seqtk in all operations where input data must be processed in multiple passes, since this data is held in memory. I expect this feature to be of great benefit when working with very large data sets. I would suggest the authors explicitly state in their experimental study which seqtk operations require multiple passes.

****RESPONSE*****

We did not use the multiple passes option in any of our experimental tests with seqkit. Note that this parameter reduces noticeably the performance of seqkit, so we have chosen not to use it to ensure a fair comparison with our tool. It is important to highlight that not all the seqkit commands support the ”two-pass” option. In our case, only sample and sort. For our new largest dataset D6, sample can still be executed without this parameter (see Table 7). On the other hand, the sort operation in seqkit cannot be executed with D6 due to memory issues even using the ”two-pass” argument.

Following the suggestion of the reviewer, the revised manuscript includes the fol.lowing sentence (page 6): ”Note that the ”two-pass” argument of seqkit was not used in the experiments.”

###################
###################

-To my surprise, no information was given about the overhead required to load the sequences to be processed into memory. In fact, some of the operations considered are I/O-bound and the resulting execution time is mainly due to the time required to read the sequences from disk to memory and vice versa. Is the load time included in the results reported by the authors?

****RESPONSE*****

Execution times for all the tools considered (BigSeqKit, seqkit, samtools and pyfastx) include the overhead of loading sequences into memory and the subsequent writing of results to disk.

Now the revised manuscript includes specifically that information (page 6).

###################

###################

In the IgnisHPC scenario, does each computational unit read a portion of the input files itself or are they loaded by a driver application and then distributed across the distributed system?

****RESPONSE*****

Each worker reads its portion of the input files, so the I/O operation is performed in parallel. There is one worker per computing node. Within each worker, its portion of the file is further divided among the available threads, improving the overall I/O performance.

Now the revised manuscript includes specifically that explanation (”Another implementation details” section -page 4).

###################
###################

In addition, the authors used an Infiniband-connected HPC infrastructure for their experiment. Do they use a remote storage server that exports a file system to all nodes of the distributed system? And, when using BigSeqKit to analyze very large files on all processing cores of a workstation, is there a potential performance/I/O bottleneck due to the controller’s limited bandwidth?

****RESPONSE*****

Our experiments were conducted using the Infiniband-connected HPC infrastructure at CESGA (Galicia Supercomputing Center, Spain). Within this infrastructure, a distributed Lustre file system is employed. It is a common practice to have dedicated storage nodes that handle the storage operations separately from the computational nodes. The Lustre system at CESGA is designed with data distribution and replication techniques to enhance performance and ensure data availability.

Now the revised manuscript explains that Lustre was used as distributed file system (page 6).

The reviewer is right that could be a potential bottleneck in the I/O performance due to the limited bandwidth of the memory controller when processing very large files. This could happen when executing commands with a very low ratio of operations per sequence. For example, the seq command. However, based on our experimental findings, that scenario is not happening since for all the commands and datasets considered, the scalability within a computing node is good.

——————————————————————————-

Reviewer #2:

This paper provides a novel parallel toolkit named BigSeqKit to manipulate FASTA and FASTQ files. BigSeqKit takes advantage of the IgnisHPC to run on the distributed and local environment. And It takes advantage of the distributed performance of IgnisHPC to optimize various operations of seqkit, and provides some new functions. Moreover, it solves the data dependency problem of some commands in a distributed environment. BigSeqKit is tens to hundreds of times faster than several state-of-the-art tools. At the same time, BigSeqKit is easy to use and install on any kind of hardware platform (local server or cluster), and its routines can be used as a bioinformatics library or from the command line.

###################
###################

Questions:
1. In Figure 4. the locate operation is an independent operation according to the paper. But in D4, with 256 cores, why did it only achieve a 50x speedup?

****RESPONSE*****

The speedup is not higher due to a small fraction of the locate routine that should be executed sequentially. Amdahl's law states that the overall speedup is limited by the proportion of the program or task that cannot be parallelized, even if the parallelizable portion is improved significantly. In other words, the impact of optimizing a specific part of a system is limited by the non-parallelizable components. For instance, if only 1.5% of the code is sequential (that is, 98.5% executed in parallel), the theoretical maximum speedup achievable using 256 cores would be 53.. Note that this percentage varies depending of the dataset.
Now the revised manuscript includes this information in pages 6-7: ”Note that speedups of some routines are not higher when using 256 cores due to there is a small fraction of the code that should be executed sequentially (Amdahl's law)”.

###################
###################

2. Different data of the same type have very different speedups, for example, locate operation on dataset D1 and D3, can you explain why?

****RESPONSE*****

The differences in speedup between the locate operation on datasets D1 and D3 can be attributed to the characteristics of the datasets. D1 consists of 1.2 million sequences ranging from 85 to 19.7K in length, while D3 has only 639 sequences ranging from 970 to 248.9M in length (see page 6 in the revised manuscript). When processing both datasets in parallel, especially when the number of cores is high, it is difficult to find a good load balance between threads when the number of sequences is low and they are of very different size (up to 248.9M bases). That is the case of D3, and the reason why the speedups are different.

Now the revised paper includes this information in page 10: ”Finally, we must high.light that one of the main reasons for the differences in the speedups between datasets running the same command with BigSeqKit is the load balance between threads. It will depend on the characteristics of the dataset: number of sequences and their length.”.

###################
###################

3. How BigSeqKit ensure the integrity of the division data? For example, how to solve if a FASTQ sequence is divided into two partitions?

****RESPONSE*****

Each worker reads its portion of the input files, so the I/O operation is performed in parallel. There is one worker per computing node. Within each worker, its portion of the file is further divided among the available threads, improving the overall I/O performance.
In a text file, the separator is represented by ’\n’, while in FASTA and FASTQ files, it is ’\n¿’ and ’\n@’, respectively. If a thread begins reading its assigned portion and does not encounter the separator, it will ignore the entire input until the separator is found. Furthermore, if a thread has completed processing its portion, it will continue reading until the separator is encountered. This approach ensures a fully parallel and coordinated reading of the input file across multiple processes and threads, as specified by the user.

Now the revised manuscript includes specifically a summary of that explanation (”An.other implementation details” section -page 4).

###################
###################

4. In the conclusion section, the authors say: ”Considering an 8-nodes cluster, BigSeqKit is even faster, reaching speedups higher than 100.”, but only one data reaches speedup over 100x, why?

Following the suggestion of the first reviewer, we have we have extended our experimental evaluation including two larger datasets with the following characteristics (page 6):
• D5 (uniprot_trembl - FASTA - 104 GB): Number of sequences: 229.9M, Minimum length: 7, Average length: 351.6, Maximum length: 45.3K.
• D6 (DRR002180 2 - FASTQ - 395 GB): Number of sequences: 1.625B, Minimum length: 101, Average length: 101, Maximum length: 101.

In the original manuscript, D5 was only used with the faidx routine. Note that D6 is larger than the memory of one computing node (395 GB vs. 256 GB).

As a result for all the commands considered and the new very large datasets, BigSeqKit is again the fastest tool. In addition, speedups are higher as data size grows, both considering 1, 2, 4 and 8 computing nodes. In particular, BigSeqKit is 144., 89.5., 159.8., 48.2., 101.1., 169.7. and 131.1. faster than the sequential execution when considering D6 and faidx, locate, replace, rmdup, sample, seq and sort commands, respectively. It means that in 5 of 7 routines achieves speedups higher than 100.

New performance results were added to Tables 3, 4, 5, 6, 7, 8 and 9, and the discussion about them is in pages 7, 8, 9 and 10 (changes highlighted in red in the revised manuscript). Conclusions (page 10) were also modified to reflect those results.

——————————————————————————–

Editor:

In addition, please register any new software application in the bio.tools and SciCrunch.org databases to receive RRID (Research Resource Identification Initiative ID) and biotoolsID identifiers, and include these in your manuscript. Computational workflows should be regis.tered in workflowhub.eu and the DOIs cited in the relevant places in the manuscript. These will facilitate tracking, reproducibility and re-use of your tool.

****RESPONSE*****

Following the suggestion of the editor, BigSeqKit was registered in bio.tools and SciCrunh.org. Both IDs (and their corresponding links) were added to the repository information in the revised manuscript (page 10):
• BiotoolsID: biotools:bigseqkit
• RRID: SCR_023592

Close
