# Supplementary material for: BigSeqKit: a parallel Big Data toolkit to process FASTA and FASTQ files at scale
Source: Gigascience. 2023 Jul 31;12:giad062. doi: 10.1093/gigascience/giad062 (PMC10388699; doi:10.1093/gigascience/giad062)
Supplement: giad062_Response_to_Reviewer_Comments_Revision_2 [file giad062_response_to_reviewer_comments_revision_2.pdf]

Author's Response To Reviewer Comments

Close

We would like to thank the reviewers and editors for their insightful comments and suggestions about the paper.

Close
