# Supplementary material for: BigSeqKit: a parallel Big Data toolkit to process FASTA and FASTQ files at scale
Source: Gigascience. 2023 Jul 31;12:giad062. doi: 10.1093/gigascience/giad062 (PMC10388699; doi:10.1093/gigascience/giad062)
Supplement: giad062_Reviewer_1_Report_Original_Submission — Umberto Ferraro Petrillo -- 4/11/2023 Reviewed [file giad062_reviewer_1_report_original_submission.pdf]

Reviewer Report

Title: BigSeqKit: a parallel Big Data toolkit to process FASTA and FASTQ files at scale

Version: Original Submission **Date:** 4/11/2023

Reviewer name: Umberto Ferraro Petrillo

Reviewer Comments to Author:

The manuscript addresses the problem of processing and manipulating large amounts of sequencing data stored in FASTA and FASTQ files. Based on the observation that most processing tools take a sequential approach, the authors present BigSeqKit, a parallelized and optimized toolkit that can be used on various hardware platforms and is tens to hundreds of times faster than other modern tools. It is described as a comprehensive and user-friendly toolkit for processing and manipulating large FASTA and FASTQ files. The paper also includes the results of experiments showing the superior performance of BigSeqKit compared to seqkit, its sequential counterpart, and other tools when a large number of processing kernels are used.Indeed, despite their very simple and inefficient structure, the FASTA and FASTQ file formats are still very common and will not be completely replaced by anything else in the foreseeable future. Against this background, the contribution of this paper might be of interest. However, I am not sure that the problem of speeding up traditional processing tools is as dramatic as the authors claim.A time saving of about 8 minutes for sorting the D3 dataset thanks to the use of 256 cores may not be so dramatic if the other steps of the analysis pipeline take hours or days, as can be the case for sequence alignments. That being said, I think the authors should provide a more solid justification for their contribution. This includes discussing, or at least anticipating, an application scenario where conventional tools fail in the first place and their approach is then needed.I have then some more punctual remarks:- After a short review of existing FASTA/Q manipulation tools, the authors conclude that none of these tools is well fitted for the manipulation of large files of tens of GB. Why? As far as I can see, the same datasets used by the authors for their experiments are even larger than one hundred GB, however the authors have been able to process them using these tools.- The paper gives the impression that BigSeqKit uses (at least) some of the code that implements seqtk. However, it is unclear how this integration is done. Is seqtk executed as a child process in the BigSeqKit tasks, or has it been integrated at the source code or library level?- The authors say that the use of IgnisHPC partitions makes it possible to improve seqtk in all operations where input data must be processed in multiple passes, since this data is held in memory. I expect this feature to be of great benefit when working with very large data sets. I would suggest the authors explicitly state in their experimental study which seqtk operations require multiple passes.- To my surprise, no information was given about the overhead required to load the sequences to be processed into memory. In fact, some of the operations considered are I/O-bound and the resulting execution time is mainly due to the time required to read the sequences from disk to memory and vice versa. Is the load time included in the results reported by the authors? In the IgnisHPC scenario, does each computational unit read a portion of the input files itself or are they loaded by a driver application and then distributed across the distributed system? In addition, the authors used an Infiniband-connected HPC infrastructure for their experiment. Do they use a remote

storage server that exports a file system to all nodes of the distributed system? And, when using BigSeqKit to analyze very large files on all processing cores of a workstation, is there a potential performance/I/O bottleneck due to the controller's limited bandwidth?

Level of Interest

Please indicate how interesting you found the manuscript: Choose an item.

Quality of Written English

Please indicate the quality of language in the manuscript: Choose an item.

Declaration of Competing Interests

Please complete a declaration of competing interests, considering the following questions:

- Have you in the past five years received reimbursements, fees, funding, or salary from an organisation that may in any way gain or lose financially from the publication of this manuscript, either now or in the future?
- Do you hold any stocks or shares in an organisation that may in any way gain or lose financially from the publication of this manuscript, either now or in the future?
- Do you hold or are you currently applying for any patents relating to the content of the manuscript?
- Have you received reimbursements, fees, funding, or salary from an organization that holds or has applied for patents relating to the content of the manuscript?
- Do you have any other financial competing interests?
- Do you have any non-financial competing interests in relation to this paper?

If you can answer no to all of the above, write 'I declare that I have no competing interests' below. If your reply is yes to any, please give details below.

I declare that I have no competing interests

I agree to the open peer review policy of the journal. I understand that my name will be included on my report to the authors and, if the manuscript is accepted for publication, my named report including any attachments I upload will be posted on the website along with the authors' responses. I agree for my report to be made available under an Open Access Creative Commons CC-BY license (http://creativecommons.org/licenses/by/4.0/). I understand that any comments which I do not wish to be included in my named report can be included as confidential comments to the editors, which will not be published.

Choose an item.

To further support our reviewers, we have joined with Publons, where you can gain additional credit to further highlight your hard work (see: https://publons.com/journal/530/gigascience). On publication of this paper, your review will be automatically added to Publons, you can then choose whether or not to claim your Publons credit. I understand this statement.

Yes Choose an item.
