# Supplementary material for: BigSeqKit: a parallel Big Data toolkit to process FASTA and FASTQ files at scale
Source: Gigascience. 2023 Jul 31;12:giad062. doi: 10.1093/gigascience/giad062 (PMC10388699; doi:10.1093/gigascience/giad062)
Supplement: giad062_Reviewer_2_Report_Original_Submission — Weiguo Liu -- 4/12/2023 Reviewed [file giad062_reviewer_2_report_original_submission.pdf]

Reviewer Report

Title: BigSeqKit: a parallel Big Data toolkit to process FASTA and FASTQ files at scale

Version: Original Submission **Date:** 4/12/2023

Reviewer name: Weiguo Liu

Reviewer Comments to Author:

This paper provides a novel parallel toolkit named BigSeqKit to manipulate FASTA and FASTQ files. BigSeqKit takes advantage of the IgnisHPC to run on the distributed and local environment. And It takes advantage of the distributed performance of IgnisHPC to optimize various operations of seqkit, and provides some new functions. Moreover, it solves the data dependency problem of some commands in a distributed environment. BigSeqKit is tens to hundreds of times faster than several state-of-the-art tools. At the same time, BigSeqKit is easy to use and install on any kind of hardware platform (local server or cluster), and its routines can be used as a bioinformatics library or from the command line.Questions:1. In Figure 4. the locate operation is an independent operation according to the paper. But in D4, with 256 cores, why did it only achieve a 50x speedup?2. Different data of the same type have very different speedups, for example, locate operation on dataset D1 and D3, can you explain why?3. How BigSeqKit ensure the integrity of the division data? For example, how to solve if a FASTQ sequence is divided into two partitions?4. In the conclusion section, the authors say: "Considering an 8-nodes cluster, BigSeqKit is even faster, reaching speedups higher than 100Ã—", but only one data reaches speedup over 100x, why?

Level of Interest

Please indicate how interesting you found the manuscript: Choose an item.

Quality of Written English

Please indicate the quality of language in the manuscript: Choose an item.

Declaration of Competing Interests

Please complete a declaration of competing interests, considering the following questions:

- Have you in the past five years received reimbursements, fees, funding, or salary from an organisation that may in any way gain or lose financially from the publication of this manuscript, either now or in the future?
- Do you hold any stocks or shares in an organisation that may in any way gain or lose financially from the publication of this manuscript, either now or in the future?
- Do you hold or are you currently applying for any patents relating to the content of the manuscript?

- Have you received reimbursements, fees, funding, or salary from an organization that holds or has applied for patents relating to the content of the manuscript?
- Do you have any other financial competing interests?
- Do you have any non-financial competing interests in relation to this paper?

If you can answer no to all of the above, write 'I declare that I have no competing interests' below. If your reply is yes to any, please give details below.

I declare that I have no competing interests

I agree to the open peer review policy of the journal. I understand that my name will be included on my report to the authors and, if the manuscript is accepted for publication, my named report including any attachments I upload will be posted on the website along with the authors' responses. I agree for my report to be made available under an Open Access Creative Commons CC-BY license (http://creativecommons.org/licenses/by/4.0/). I understand that any comments which I do not wish to be included in my named report can be included as confidential comments to the editors, which will not be published.

Choose an item.

To further support our reviewers, we have joined with Publons, where you can gain additional credit to further highlight your hard work (see: https://publons.com/journal/530/gigascience). On publication of this paper, your review will be automatically added to Publons, you can then choose whether or not to claim your Publons credit. I understand this statement.

Yes Choose an item.
